# N_2_ Gas Flushing Alleviates the Loss of Bacterial Diversity and Inhibits Psychrotrophic *Pseudomonas* during the Cold Storage of Bovine Raw Milk

**DOI:** 10.1371/journal.pone.0146015

**Published:** 2016-01-05

**Authors:** Silvia Gschwendtner, Tapani Alatossava, Susanne Kublik, Mirna Mrkonjić Fuka, Michael Schloter, Patricia Munsch-Alatossava

**Affiliations:** 1 Research Unit for Environmental Genomics, Helmholtz Zentrum München, Neuherberg, Germany; 2 Department of Food and Environmental Sciences, University of Helsinki, Helsinki, Finland; 3 Department of Microbiology, Faculty of Agriculture, University of Zagreb, Zagreb, Croatia; Agricultural University of Athens, GREECE

## Abstract

The quality and safety of raw milk still remains a worldwide challenge. Culture-dependent methods indicated that the continuous N_2_ gas-flushing of raw milk reduced the bacterial growth during cold storage by up to four orders of magnitude, compared to cold storage alone. This study investigated the influence of N_2_ gas-flushing on bacterial diversity in bovine raw-milk samples, that were either cold stored at 6°C or additionally flushed with pure N_2_ for up to one week. Next-generation sequencing (NGS) of the V1-V2 hypervariable regions of 16S rRNA genes, derived from amplified cDNA, which was obtained from RNA directly isolated from raw-milk samples, was performed. The reads, which were clustered into 2448 operational taxonomic units (OTUs), were phylogenetically classified. Our data revealed a drastic reduction in the diversity of OTUs in raw milk during cold storage at 6°C at 97% similarity level; but, the N_2_-flushing treatment alleviated this reduction and substantially limited the loss of bacterial diversity during the same cold-storage period. Compared to cold-stored milk, the initial raw-milk samples contained less Proteobacteria (mainly Pseudomonadaceae, Moraxellaceae and Enterobacteriaceae) but more Firmicutes (mainly Ruminococcaceaea, Lachnospiraceae and Oscillospiraceaea) and Bacteroidetes (mainly Bacteroidales). Significant differences between cold-stored and additionally N_2_-flushed milk were mainly related to higher levels of Pseudomononadaceae (including the genera *Pseudomonas* and *Acinetobacter*) in cold-stored milk samples; furthermore, rare taxa were better preserved by the N_2_ gas flushing compared to the cold storage alone. No major changes in bacterial composition with time were found regarding the distribution of the major 9 OTUs, that dominated the *Pseudomonas* genus in N_2_-flushed or non-flushed milk samples, other than an intriguing predominance of bacteria related to *P*. *veronii*. Overall, this study established that neither bacteria causing milk spoilage nor any well-known human pathogen or anaerobe benefited from the N_2_ gas flushing even though the N_2_-flushed and non-flushed cold-stored milk differed in bacterial counts by up to 10^4^-fold.

## Introduction

The microbiological quality of raw milk and of dairy products is primarily determined by the initial level and composition of the bacterial population present in raw milk. Current existing international and national legislations have set upper limits for bovine raw milk in terms of acceptable microbiological quality as the “total” bacterial counts per ml in the tank at the farm and at the dairy silo level, at 10^5^ cfu/ml and 3x10^5^ cfu/ml, respectively, when the milk samples are cultured on standard Plate Count Agar (PCA) for 3 days at 30°C under aerobic conditions; these bacterial levels correspond to the rolling geometric average over a two-month period, with at least two samples per month [1].

To maintain the microbiological quality of the raw milk produced on a farm, the current legislation in developed countries requires the storage of raw milk at a temperature of 6°C or less (usually at 2 to 4°C) soon after milking. The chill-chain of raw-milk storage and transport relies on the transfer of milk from the farm bulk tank to a tank on a truck transfer to the dairy silo where the milk is further processed. During cold storage at 4°C, the “total” bacterial counts of fresh bovine raw milk can be kept constant for approximately two days, after which the psychrotrophs (defined as those microbes able to grow at 7°C), which are mainly pseudomonads, rapidly increase and become the predominant population among the raw-milk microbes [2, 3, 4, 5, 6]. Numerous studies have investigated the effect of cold storage on the microbial community composition using traditional cultivation-based approaches [6, 7, 8], or molecular methods based on direct DNA extraction from milk samples [9, 10, 11].

Using cultivation-based methods, we previously observed that continuous flushing with pure N_2_ gas showed some potential for alleviating bacterial spoilage of raw milk in the laboratory and at a pilot plant scale because the growth of mesophiles, psychrotrophs, lipase and protease producers was impeded, but anaerobes, lactic acid bacteria, or enterobacteria were not favored [12, 13]. Thus, such treatment could provide the possibility to preserve the microbiological quality and the organoleptic properties of raw milk, and could also serve to extend the cold-storage time of raw milk before dairy processing. However so far, only cultivation-based approaches have been used to investigate the consequences of N_2_ treatment on bacterial diversity, which do not allow for conclusions to be drawn about the effects of the treatment on the overall bacterial diversity.

Consequently, the purpose of the present study was to describe the bacterial diversity of bovine raw-milk samples using a direct molecular barcoding approach without prior cultivation and to investigate whether continuous N_2_ gas flushing of cold-stored raw milk modifies the bacterial community structure. As several studies have indicated, DNA-based approaches might be biased as DNA can persist for some time even after the host bacteria have died [14]. We used rRNA as the target to analyze the bacterial community composition. Furthermore, for many bacteria the rRNA content is also related to their activity [15], which may be an important additional parameter when the impact on milk spoilage is considered.

## Materials and Methods

### Raw-milk samples and microbiological analyses

Three different bovine raw-milk samples (L1, L2, and L3) were considered; they represented comingled milks delivered by trucks to the Helsinki Dairy Ltd. in Helsinki (Finland), in May, June and August 2014. The N_2_ gas flushing treatment and the microbiological analyses were performed as previously described [12]. The N_2_ gas (AGA Ltd, Riihimäki, Finland) was 99.999% pure; the flow rate was adjusted to 120ml/min; sterile filtered gas entered constantly the headspace of a test bottle that contained 100 ml of raw milk; both the control (C) and the N_2_-flushed milk (N) were continuously mixed while stored at 6°C. At the appropriate sampling times, the milk was serially diluted in 0.85% saline solution. “Total” aerobic bacterial counts were determined from triplicate PCA platings after incubation at 30°C for 72 h.

The description of the milk samples L1, L2 and L3, is as follows: N and C indicate whether the milk was flushed (N) or not (C), respectively; the numbers which follow N or C depict the time of the analyses and correspond to the number of days elapsed during cold storage.

### RNA extraction and amplicon sequencing

After receipt of the raw-milk samples and after 3, 4, 6 or 7 days of cold storage, 1.5 ml of raw milk was withdrawn from the cold-stored or cold-stored N_2_-flushed milk bottles. RNA was directly extracted using the RNeasy kit (Qiagen, Sweden), according to the manufacturer´s guidelines. The RNA extract was subjected to DNase I treatment (RNase-Free DNase set, Qiagen, Sweden). The Sensi FAST ^TM^ cDNA synthesis kit (Bioline, Biotop Oy, Turku, Finland) was used for cDNA synthesis according to the manufacturer´s guidelines. A control PCR targeting a 0.7 kb fragment of the 16S rRNA gene was performed with the primers WO1 and WO12 [16], to ensure that no bacterial DNA was present and that cDNA synthesis from each of the RNA extracts was successful, prior to further study.

Amplicon next-generation sequencing (NGS) was performed on a MiSeq Illumina (Illumina, United Kingdom, Chesterford). The universal eubacterial primers 27f (5’- *TCGTCGGCAGCGTCAGATGTGTATAAGAGACAG-*AGAGTTTGATCMTGGC-3’) and 357r (5’- *GTCTCGTGGGCTCGGAGATGTGTATAAGAGACAG*-CTGCTGCCTYCCGTA-3’) covering the V1-V2 hypervariable regions of the 16S rRNA gene [17] were extended with their respective overhangs (in italics) to match the Illumina indexing primers. Sequencing PCR was performed as follows: initial denaturation (98°C, 5 min), followed by 25 cycles of denaturation (98°C, 10 s), annealing (60°C, 30 s) and elongation (72°C, 30 s), ending with a final extension (72°C, 5 min). Each 25-μl PCR reaction contained 1x NebNext High Fidelity Mastermix (New England Biolabs, Germany), 0.5 mM of each primer and 1 ng of template DNA. PCR products were visualized on 1% agarose gels to verify the product size and purified twice using AMPure beads XP (Beckman Coulter, Germany). After measuring the fragment size and concentration with a Bioanalyzer 2100 device on a High Sensitivity DNA Chip (Agilent, Germany), index PCR was performed under the following conditions: 98°C for 5 min, followed by 8 cycles of 98°C for 10 s, 55°C for 30 s and 72°C for 30 s, and final extension at 72°C for 5 min. Each 25-μl PCR reaction contained 1x NebNext High Fidelity Mastermix (New England Biolabs, Germany), index primer 1 (N7xx) and index primer 2 (S5xx) as well as 2.5 μl template DNA, according to manufacturer’s instructions. All samples were purified using AMPure beads XP (Beckman Coulter, Germany), validated using a Bioanalyzer 2100 device on a High-Sensitivity DNA Chip (Agilent, Germany) and quantified via Quant-iT Pico Green dsDNA Assay Kit (Invitrogen, Germany). Afterwards, the libraries were pooled to a final concentration of 2 nM for the MiSeq sequencing run.

### Sequence data analysis

To reduce sequencing errors, the raw sequences were processed with MOTHUR v.1.33.3 [18]. Firstly, barcodes and primers were removed to form contiguous sequences. Afterwards, the sequences were checked for chimeras by alignment to the SILVA database provided by MOTHUR, which is derived from a sequence collection of the Genomes online database [19]. Sequences were classified using the Ribosomal Database Project dataset in MOTHUR. After removing mitochondrial and chloroplastic sequences a distance matrix was calculated from the high-quality aligned sequences, resulting in operational taxonomical units (OTUs) obtained by the furthest neighbor clustering algorithm.

For the phylogenetic analysis of *Pseudomonas*, which was the genus with the highest responsiveness to the milk storage conditions, representative sequences of each OTU were clustered in MOTHUR at a 97% similarity level and were aligned to the SILVA database using ARB [20].

The nucleotide sequence data obtained in this study have been deposited to the NCBI Sequence Read Archive under accession numbers SRR2481188, SRR2481213, SRR2481233, SRR2481257, SRR2481271, SRR2481286, SRR2481304, SRR2481319, SRR2481331, SRR2481345, SRR2481360, SRR2481370, SRR2481383, SRR2481397 and SRR2481507.

### Statistical analysis

For sequence data analyses, a distance matrix based on OTUs defined by 97% similarity was generated for calculating rarefaction curves, OTU richness and diversity indices (Shannon, Pielou’s evenness). Phylogenetic analysis was also conducted at a 97% similarity level. As Principal Component Analysis revealed a strong clustering of the initial raw-milk samples (L10, L20, L30) (data not shown), even though the milk was produced in different months, the three samples were treated as replicates for statistical purposes. Significant differences according to the milk-storage conditions were analyzed in R v3.1.2 (http://www.R-project.org/) using a multivariate analysis of variance (Adonis function) based on the Yue Clayton dissimilarity index and Euclidean distances of Hellinger-transformed data [21]. For *Pseudomonas*, a maximum likelihood consensus tree was calculated from all of the aligned sequences in ARB using the default settings.

## Results

### Cultivation-based analyses of bacteria from bovine raw milk

**Fig 1:**
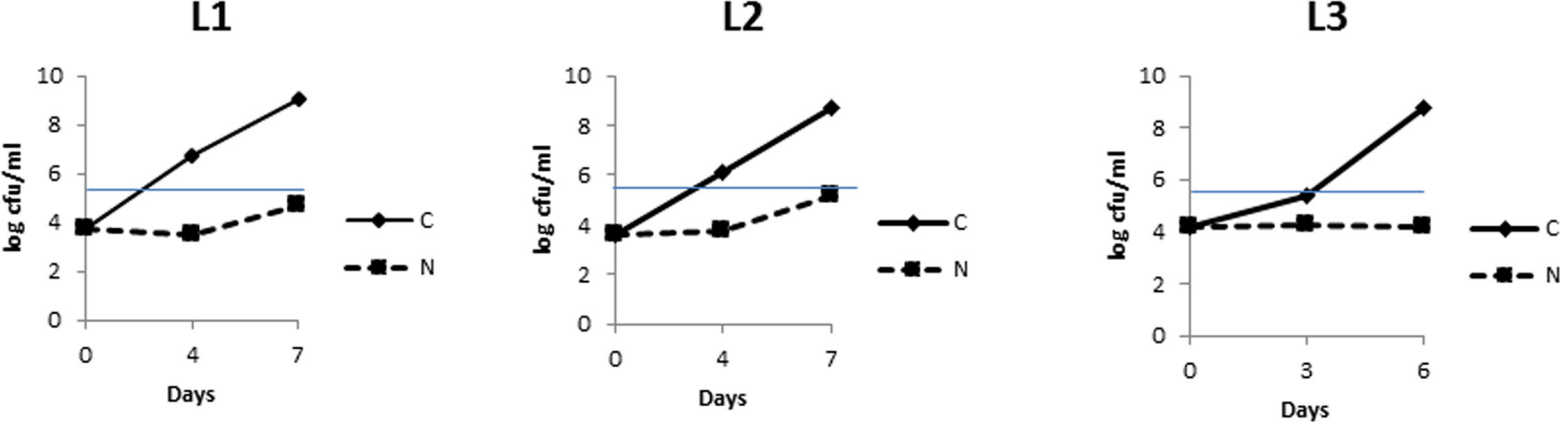
Time course analyses of the number of bacteria present in the three truckloads of bovine raw-milk samples (L1, L2, and L3), enumerated on PCA agar after 3 days incubation at 30°C under aerobic conditions: C (cold-stored milk at 6°C), N (milk flushed with N_2_ gas while in cold storage at 6°C). Error bars indicate standard deviations. The blue line corresponds to the 3x10^5^ (5.5 log-units) threshold value for raw milk acceptance for dairy processing.

Initial bacterial counts on PCA agar were approximately 10^4^ cfu/ml (approximately 4 log-units) for all three raw-milk samples (L1, L2, and L3) (Fig 1). For the controls (C), substantial growth occurred over time, as expected. For L1 and L2, the counts exceeded 10^6^ cfu/ml (6 log-units) after 4 days of cold storage, but L3 was still below 5.5 log-units at day 3. After 6 or 7 days, the bacterial counts ranged between 8 and 9 log-units, and had increased 10^4^ to 10^5^-fold compared to the initial levels. In contrast, the bacterial load remained rather constant during 3 or 4 days for the N_2_-flushed milk, with no increase after 6 days in sample L3 and with a small increase after 7 days in samples L1 and L2 (Fig 1). However, all of the final bacterial counts were still below the microbiological acceptance limit (3x10^5^ cfu/ml, equivalent to 5.5 log-units) for the N_2_-flushing treatment.

### Bacterial diversity analysis of bovine raw milk

All cDNAs synthesized from the subsamples (L10, L1C4, L1N4, L1C7, L1N7; L20, L2C4, L2N4, L2C7, L2N7; L30, L3C3, L3N3, L3C6, L3N6) yielded a 16S rRNA-PCR product of the expected size, but the PCR failed to produce an amplicon from the corresponding RNA extracts, which indicated that no DNA remained in the samples (data not shown).

**Fig 2:**
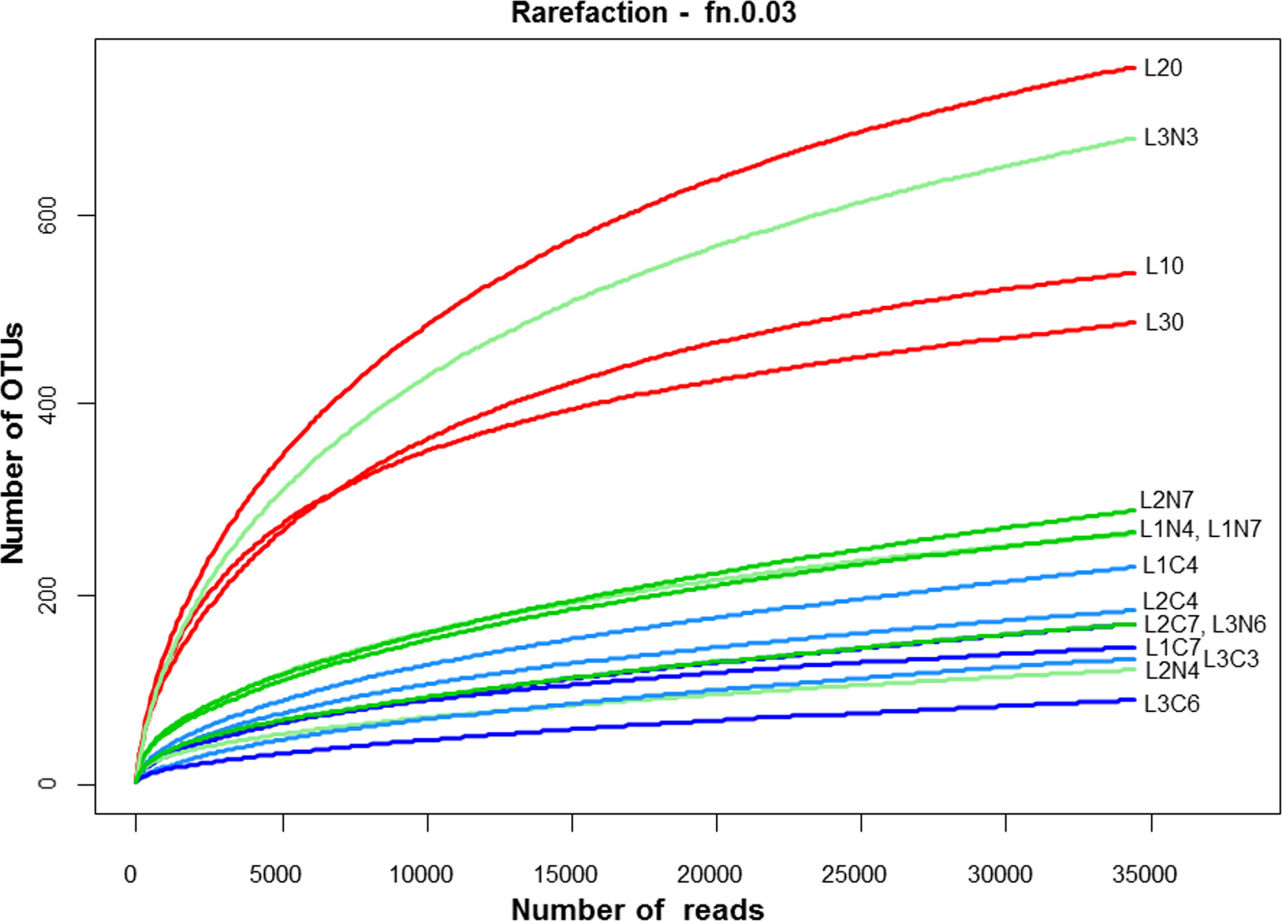
Rarefaction curves of partial 16S rRNA transcript sequences after RNA extraction, cDNA synthesis and PCR amplification from three truckloads of milk samples divided into 15 subsamples as follows: L10, L20, L30 (initial samples), cold-stored at 6°C for 3 to 4 days (L1C4, L2C4, L3C3) for 6 to 7 days (L1C7, L2C7, L3C6); cold-stored at 6°C and N_2_-flushed for 3 to 4 days (L1N4, L2N4, L3N3) for 6 to 7 days (L1N7, L2N7, L3N6).

In total, 998317 bacterial raw-sequence reads were generated from the PCR amplicons by Illumina sequencing. After noise filtering, a chimera check and the removal of erroneous reads, 773138 high-quality partial 16S rRNA gene sequences covering V1-V2 hypervariable regions with a minimum length of 300 bp remained and could be assigned to 2448 OTUs at 97% similarity. To compare samples without statistical bias, 34356 reads were chosen for all samples for the calculation of richness and diversity indices, reflecting the read number of the sample with the lowest number of reads (L3C6) obtained. The analysis of the rarefaction curves indicated that this sampling depth was sufficient for further analysis of samples at an OTU_97_ level because plateaus were reached for all samples (Fig 2). Overall, bacterial richness was highest in initial raw milk (L10, L20, L30), ranging from 486 to 756 OTUs. In all other samples, bacterial richness was reduced and the number of OTUs ranged from 89 (L3C6) to 288 (L2N7) per sample. The only exception was L3N3 for which a diversity level comparable to the initial raw-milk samples was detected (Fig 2).

Coincidentally, bacterial diversity, as calculated via Shannon index, and bacterial evenness were highest in the initial raw-milk samples (3.47 and 0.55, respectively) and lowest in the cold-stored samples (1.29 and 0.25, respectively) (Table 1).

**Table 1:** OTU richness and diversity indices for bacterial communities in initial raw-milk samples, in milk cold-stored at 6°C, and in milk cold-stored while N_2_-flushed for 3 to 4 or 6 to 7 days (n = 3, standard deviation in parentheses). Significant differences were calculated using a multivariate ANOVA and are indicated by p values <0.05 (bold characters).

Conditions	Time (days)	No. OTUs	Shannon	Evenness
**Initial raw milk**	-	594 (143)	3.47 (0.05)	0.55 (0.02)
**Cold-stored**	3 to 4	181 (48)	1.36 (0.76)	0.26 (0.14)
**Cold-stored**	6 to 7	134 (41)	1.22 (0.48)	0.25 (0.08)
**Cold-stored**	3 to 4 and 6 to 7	158 (48)	1.29 (0.57)	0.25 (0.10)
**Cold-stored +N**_**2**_ **-flushed**	3 to 4	355 (290)	2.22 (0.78)	0.39 (0.08)
**Cold-stored +N**_**2**_ **-flushed**	6 to 7	241 (63)	2.22 (0.92)	0.40 (0.15)
**Cold-stored +N**_**2**_ **-flushed**	3 to 4 and 6 to 7	298 (198)	2.22 (0.76)	0.39 (0.11)
**p**_**milk storage**_		0.001	0.001	0.006
**p**_**time**_		0.302	0.302	0.371
**p**_milk storage * time_		0.192	0.192	0.243

When comparing both types of cold storage with or without N_2_-flushing, a significantly higher number of OTUs were found in the cold-stored flushed raw-milk samples, compared to the cold- stored alone (Table 1).

**Fig 3:**
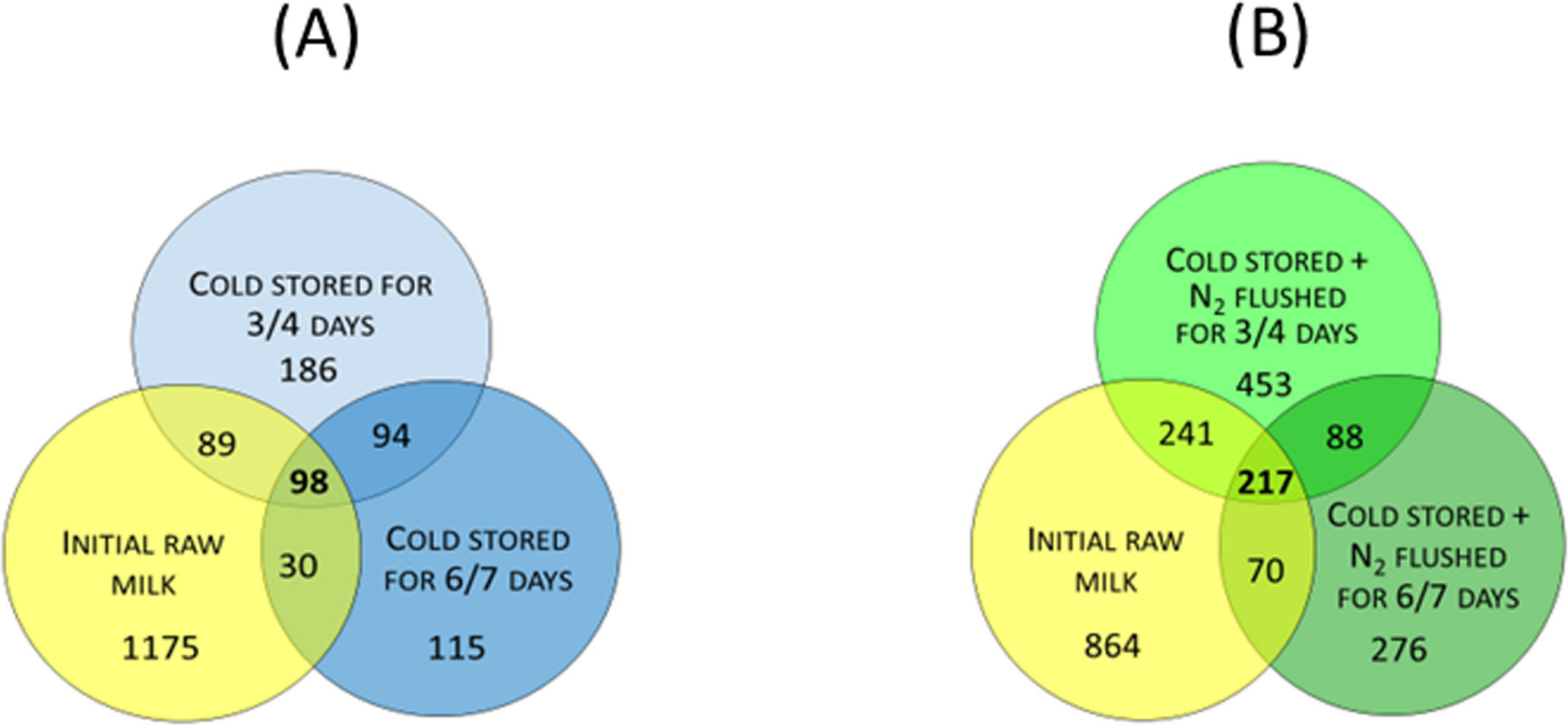
Venn diagrams of Illumina sequence data comparing (A) initial and cold-stored raw-milk samples (B) initial and cold-stored N_2_-flushed raw-milk samples. OTUs shared between the conditions are indicated in boldface.

Under cold storage alone, the total OTU number decreased from 1392 to 467 after 3 to 4 days (66.5% loss) and to 337 after 6 to 7 days (75.8% loss) (Fig 3A). After 6 to 7 days, the cold-stored samples shared only 128 OTUs (37.9%) with the initial raw milk. Moreover, only 98 OTUs (7.0%), present at the initial stage, were preserved during the entire cold-storage period at 6°C (Fig 3A).

Cold storage combined with the N_2_-gas-flushing treatment attenuated the reduction of OTU numbers as after 3 to 4 and 6 to 7 days, respectively 999 and 651 OTUs could be still detected which was equivalent to a loss of only 28.2 and 53.2% of the OTUs, respectively (Fig 3B). After 6 to 7 days, the cold-stored N_2_-flushed samples still shared 287 OTUs (44.1%) with the initial raw-milk samples; up to 217 OTUs (15.6%) were recovered in both the flushed and initial raw-milk samples (Fig 3B).

**Fig 4:**
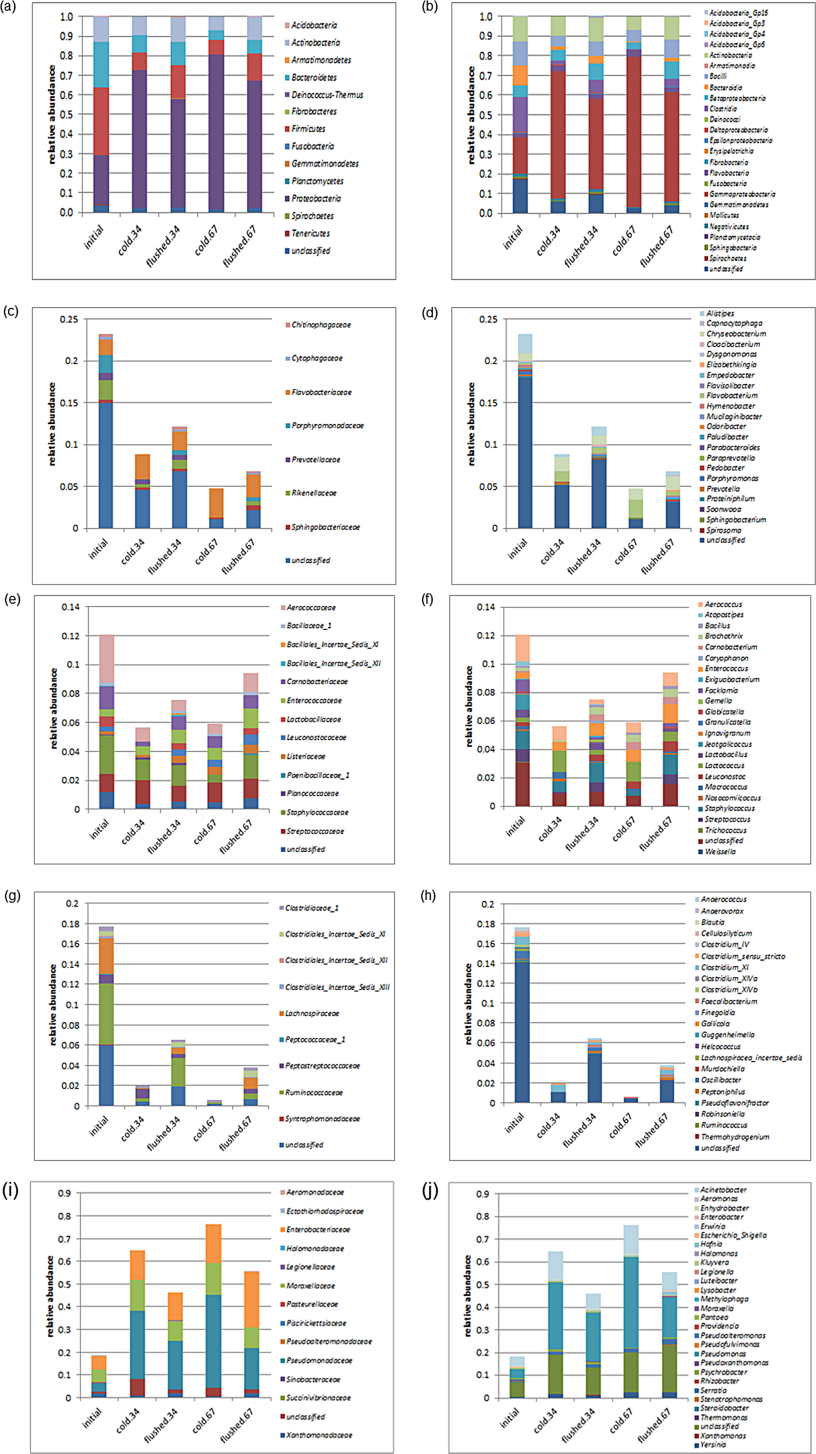
Composition of the bacterial communities based on 16S rRNA transcripts in initial raw milk, in cold-stored milk, and in cold-stored while N_2_-flushed for either 3 or 4 and 6 or 7 days, based on partial 16S rRNA gene sequences after RNA extraction, cDNA synthesis and PCR amplification. (a) Total phyla, (b) total classes, (c) families of Bacteroidetes, (d) genera of Bacteroidetes, (e) families of Bacilli, (f) genera of Bacilli, (g) families of Clostridia, (h) genera of Clostridia, (i) families of Gammaproteobacteria, (j) genera of Gammaproteobacteria.

In total, 13 phyla were detected: *Acidobacteria*, *Actinobacteria*, *Armatimonadetes*, *Bacteroidetes*, *Deinococcus-Thermus*, *Fibrobacteres*, *Fusobacteria*, *Gemmatimonadetes*, *Planctomycetes*, *Proteobacteria*, *Spirochaetes*, *Tenericutes* and *Firmicutes* (Fig 4A, S1 Table). More than 80% of the annotated reads were grouped into three major phyla, *Bacteroidetes*, *Proteobacteria* and *Firmicutes*, which ranked *Firmicutes* > *Proteobacteria* ≈ *Bacteroidetes* in the initial raw milk, *Proteobacteria* >> *Bacteroidetes* ≈ *Firmicutes* in the cold-stored milk, and *Proteobacteria* > *Firmicutes* > *Bacteroidetes* in cold-stored N_2_-flushed samples (Fig 4A).

Bacteroidetes-related rRNA was dominated by members of Bacteroidia. While accounting for 10.2% of total reads in the initial raw-milk samples, after 7 days, the Bacteroidia rRNA decreased to 0.2 and 1.7% in the milk samples that were only cold-stored and in the samples that were cold-stored and N_2_-flushed, respectively (Fig 4B, S1 Table). In total, seven Bacteroidetes families were detected (Fig 4C). In the initial raw-milk samples, Flavobacteriaceae, Porphyromonadaceae and Rikenellaceae were evenly distributed, accounting for 1.8–2.3% of all reads, but only Flavobacteriaceae remained constant during the milk storage while the others decreased. Flavobacteriaceae were dominated by the genera *Flavobacterium* and *Chryseobacterium*, comprising 56.5–95.8% of this family (Fig 4D), while 23.6–77.5% of Bacteroidetes could not be classified to the genus level.

Bacilli and Clostridia were the major groups of Firmicutes (Fig 4B, S1 Table). Both classes were most prevalent in the initial raw-milk samples, accounting for 12.1% and 17.7% of the total reads. While Bacilli rRNA was only temporarily reduced after 3 to 4 days of cold storage, Clostridia rRNA decreased significantly during milk storage with more pronounced effects in cold storage without N_2_ flushing.

For Bacilli, 13 families were detected (Fig 4E). In the initial raw-milk samples, *Aerococcaceae* (3.3%), *Staphylococcaceae* (2.7%), *Carnobacteriaceae* (1.6%) and *Streptococcaceae* (1.2%) were the principal families of Bacilli. Although slightly decreased, no significant storage effect was observed due to the high variation among replicates (Fig 4E, S1 Table). However, Staphylococcaceae belonging to the genus *Jeotgalicoccus* (1.0% of the total reads in initial raw-milk samples) significantly declined under both storage conditions (Fig 4F). Reads that could be clearly assigned to the lactic acid bacteria of interest to dairies (*Lactobacillus*, *Lactococcus*, *Streptococcus*, *Leuconostoc*) were in low abundance comprising a total of 1.6–2.3% of all reads (Fig 4F, Table 2).

**Table 2:** Relative abundance and number of OTUs of selected bacterial taxa present in initial and either cold-stored or cold-stored N_2_-flushed raw milk (n = 3, standard deviation in parentheses). The taxa listed below correspond to the most abundant bacterial groups in the initial raw-milk samples, together with taxa hosting human pathogens, dairy starters, dairy secondary cultures, milk spoilage bacteria and groups for which this study highlighted changes subsequent to the applied treatments (either cold storage at 6°C, or cold storage combined with N_2_ flushing). The complete list of bacterial taxa found in all samples is shown in S1 Table. Data of the 773138 reads of sequences with the corresponding 2448 OTUs are available under the accession numbers SRR2481188, SRR2481213, SRR2481233, SRR2481257, SRR2481271, SRR2481286, SRR2481304, SRR2481319, SRR2481331, SRR2481345, SRR2481360, SRR2481370, SRR2481383, SRR2481397 and SRR2481507.

	CONDITIONS	Initial raw milk	cold-stored	cold-stored+N_2_-flushed	cold-stored	cold-stored+N_2_-flushed	OTU no.
	Time	0 day	3 to 4 days	3 to 4 days	6 to 7 days	6 to 7 days	
		reads = 134076	reads = 155564	reads = 156150	reads = 170269	reads = 157079	
**Phylum**	**Genus**						
***Actinobacteria***	*Brevibacterium*	0.010 (0.006)	0.011 (0.006)	0.012 (0.009)	0.011 (0.005)	0.010 (0.001)	15
	*Corynebacterium*	0.026 (0.001)	0.018 (0.010)	0.021 (0.013)	0.011 (0.005)	0.017 (0.005)	46
	*Kocuria*	0.011 (0.011)	0.014 (0.004)	0.014 (0.005)	0.009 (0.004)	0.010 (0.004)	16
	*Micrococcus*	0.005 (0.002)	0.013 (0.009)	0.009 (0.003)	0.006 (0.002)	0.009 (0.002)	7
*** ***	*Propionibacterium*	0.004 (0.001)	0.007 (0.004)	0.004 (0.001)	0.005 (0.005)	0.006 (0.002)	4
***Bacteroidetes***	*Alistipes*	0.023 (0.009)	0.004 (0.007)	0.010 (0.015)	0.000 (0.000)	0.005 (0.006)	45
	*Bacteroidales_u* ^*a*^	0.049 (0.003)	0.012 (0.010)	0.018 (0.023)	0.002 (0.003)	0.007 (0.003)	75
	*Bacteroidetes_u*	0.098 (0.010)	0.034 (0.032)	0.049 (0.064)	0.009 (0.008)	0.013 (0.005)	166
*** ***	*Chryseobacterium*	0.009 (0.002)	0.016 (0.011)	0.011 (0.007)	0.014 (0.010)	0.016 (0.008)	14
***Firmicutes***	*Bacillus*	0.001 (0.001)	0.000 (0.000)	0.002 (0.002)	0.002 (0.003)	0.002 (0.003)	4
	*Clostridiales_u*	0.059 (0.021)	0.004 (0.007)	0.018 (0.027)	0.002 (0.003)	0.006 (0.008)	124
	*Clostridium* (sum) ^b^	0.014 (0.005)	0.009 (0.012)	0.004 (0.006)	0.000 (0.000)	0.008 (0.006)	17
	*Enterococcus*	0.005 (0.002)	0.006 (0.002)	0.009 (0.003)	0.008 (0.003)	0.014 (0.001)	11
	*Jeotgalicoccus*	0.010 (0.009)	0.000 (0.000)	0.000 (0.000)	0.000 (0.000)	0.000 (0.000)	14
	*Lachnospiraceae_u*	0.028 (0.005)	0.001 (0.002)	0.006 (0.006)	0.000 (0.000)	0.009 (0.008)	55
	*Lactobacillus*	0.005 (0.002)	0.000 (0.000)	0.004 (0.004)	0.000 (0.000)	0.002 (0.003)	10
	*Lactococcus*	0.004 (0.002)	0.015 (0.009)	0.004 (0.003)	0.014 (0.013)	0.007 (0.008)	7
	*Leuconostoc*	0.003 (0.001)	0.000 (0.000)	0.005 (0.003)	0.005 (0.004)	0.007 (0.008)	3
	*Oscillibacter*	0.008 (0.002)	0.000 (0.000)	0.003 (0.005)	0.000 (0.000)	0.000 (0.000)	18
	*Ruminococcaceae_u*	0.050 (0.015)	0.003 (0.003)	0.022 (0.026)	0.000 (0.000)	0.005 (0.002)	127
	*Staphylococcus*	0.013 (0.004)	0.007 (0.009)	0.014 (0.009)	0.003 (0.003)	0.014 (0.006)	15
* *	*Streptococcus*	0.009 (0.005)	0.001 (0.002)	0.007 (0.007)	0.000 (0.000)	0.007 (0.004)	14
***Proteobacteria***	*Acinetobacter*	0.042 (0.011)	0.124 (0.056)	0.069 (0.010)	0.128 (0.053)	0.079 (0.026)	111
	*Campylobacter*	0.004 (0.004)	0.002 (0.003)	0.002 (0.004)	0.000 (0.000)	0.001 (0.002)	6
	*Delftia*	0.002 (0.001)	0.005 (0.001)	0.007 (0.005)	0.003 (0.003)	0.007 (0.001)	8
	*Enterobacteriaceae_u*	0.040 (0.048)	0.091 (0.076)	0.090 (0.088)	0.121 (0.031)	0.168 (0.092)	163
	*Escherichia_Shigella*	0.001 (0.001)	0.000 (0.000)	0.000 (0.000)	0.000 (0.000)	0.000 (0.000)	1
	*Hafnia*	0.002 (0.000)	0.003 (0.002)	0.003 (0.002)	0.006 (0.002)	0.013 (0.004)	8
	*Pseudomonadales_u*	0.000 (0.001)	0.048 (0.041)	0.005 (0.001)	0.026 (0.032)	0.005 (0.005)	32
	*Pseudomonas*	0.037 (0.010)	0.297 (0.051)	0.214 (0.148)	0.401 (0.071)	0.176 (0.110)	257
	*Psychrobacter*	0.009 (0.004)	0.009 (0.008)	0.012 (0.007)	0.005 (0.004)	0.007 (0.004)	18
	*Serratia*	0.006 (0.009)	0.014 (0.013)	0.009 (0.007)	0.018 (0.004)	0.027 (0.017)	16
* *	*Yersinia*	0.005 (0.007)	0.016 (0.015)	0.013 (0.015)	0.024 (0.014)	0.025 (0.009)	17

^a^u, unclassified

^b^sum, sum of *Clostridium* sensu stricto groups (IV, XI, XIVa and XIVb)

**Fig 5:**
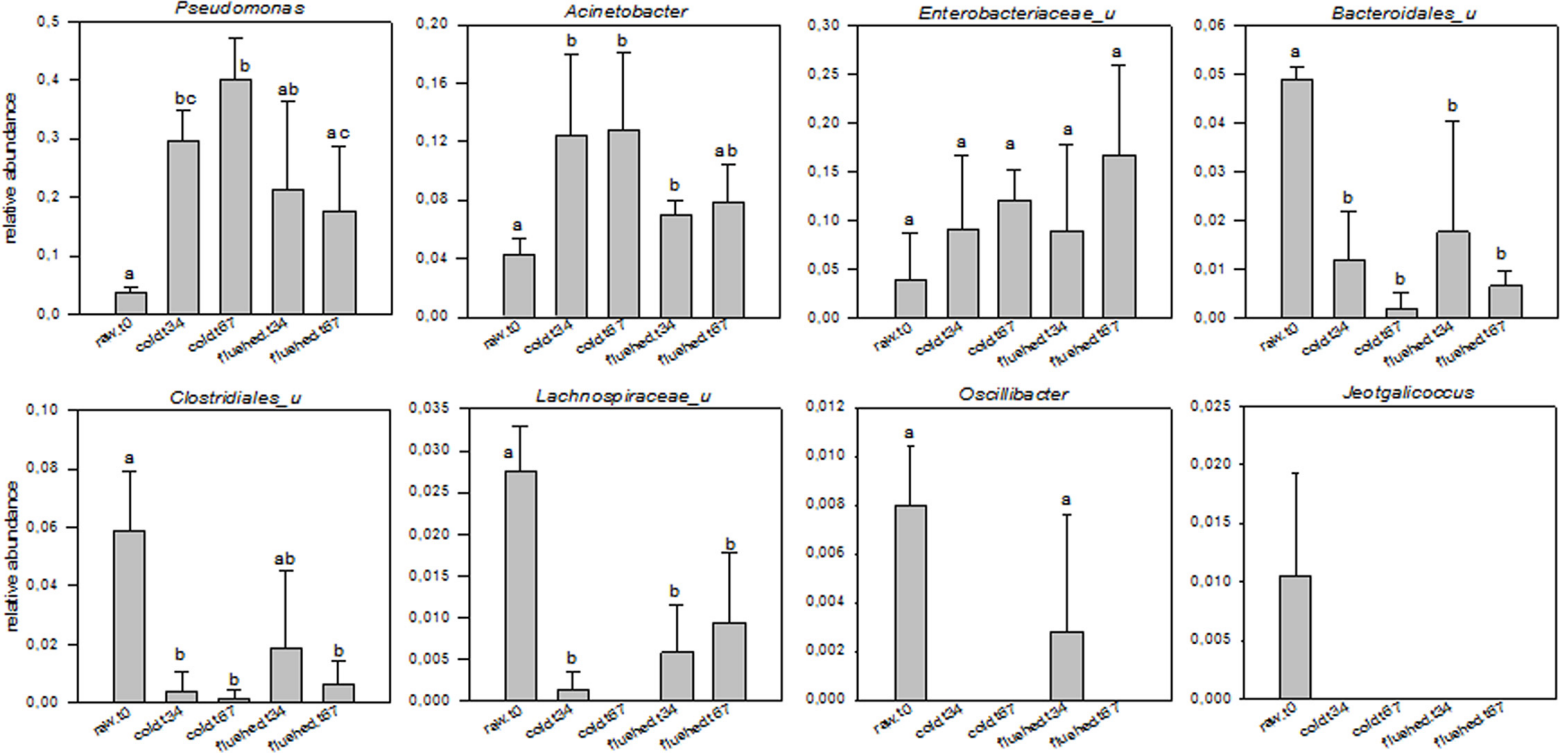
Relative abundance of raw milk taxa that were significantly affected by cold storage at 6°C and by cold storage combined with N_2_ gas flushing. Error bars represent the standard deviation of mean (n = 3) values. Average values sharing a common letter are not significantly different with a level of significance of 0.05 over all comparisons. (u_: unclassified).

Clostridia were grouped into nine families with Ruminococcaceae and Lachnospiraceae predominating (Fig 4G, S1 Table). Both decreased significantly from 6.1% and 3.4% in the initial raw- milk samples to 0.0% and 0.2% (for cold-stored milk) and to 1.1% and 0.6% (for cold-stored N_2_-flushed milk) after 6 to 7 days of milk storage (Fig 4G, Fig 5). Ruminococcaceae belonging to the genus *Oscillibacter*, which accounted for 0.8% of all reads in the initial raw-milk samples, could not be detected after 6 to 7 days of cold storage (Fig 4H, Fig 5, Table 2).

A large proportion of Clostridia (53.3–79.8%) could not be further classified at the genus level.

Proteobacteria were dominated by Gammaproteobacteria (Fig 4B, S1 Table), accounting for 18.2% of the total reads in the initial raw-milk samples. During storage, Gammaproteobacteria increased dramatically to 76.2% (in cold-stored milk) and 55.6% (for cold-stored N_2_-flushed milk). In total, 13 families were detected with Pseudomonadaceae > Enterobacteriaceae ≥ Moraxellaceae, comprising in total 65.6–85.2% of all Gammaproteobacteria (Fig 4I). While > 70% of Enterobacteriaceae could not be classified into genera using the SILVA reference database, members of *Pseudomonas* and *Acinetobacter* clearly dominated Pseudomonadaceae and Moraxellaceae; up to 99.8% of Pseudomonadaceae and 74.3–91.1% of Moraxellaceae could be classified into their respective genera (Fig 4J). Moreover, those genera were significantly affected by the milk storage conditions. After 3 to 4 days of cold storage alone, *Pseudomonas*-related rRNA increased significantly from 3.7% to 29.7% (Fig 4J, Fig 5, Table 2, S1 Table). Extended cold storage still increased the dominance of *Pseudomonas*, which constituted the major group (40.1%) after 6 to 7 days (Fig 5, Table 2, S1 Table). The rise of *Pseudomonas-*related rRNA coincided with an increase of *Acinetobacter*-related rRNA from 4.2% to 12.8% after 6 to 7 days. For N_2_-flushed samples, *Pseudomonas* rRNA increased from 3.7% to 21.4% after 3 to 4 days, although the increase was not significant because of high sample variation (Fig 4J, Fig 5, Table 2, S1 Table). To a lesser extent, *Acinetobacter* rRNA also increased after 3 to 4 days of N_2_ flushing (from 4.2% to 6.9%) (Figs 4J and 5, Table 2, S1 Table). Surprisingly, the abundance of *Pseudomonas*-related rRNA decreased after 6 to 7 days (17.6%) and was significantly lower compared to the milk cold-stored alone at the same sampling time (Fig 5).

Irrespective of the samples or the treatments, *Pseudomonas*-related rRNA was dominated (61–97%) by sequences closely related to *P*. *veronii* (Table 3, S1 Fig). Other *Pseudomonas-*related OTUs were detected more infrequently and could be assigned to *P*. *nitroreducens*, *P*. *alcaligenes*, *P*. *lini*, *P*. *fragi*, *P*. *putida*, *P*. *lundensis* or *P*. *panipatensis* (Table 3, S1 Fig).

**Table 3:** Relative abundance (%) and phylogenetic position of the nine most common OTUs among 257 OTUs related to *Pseudomonas* present in the 15 subsamples representing the initial (L10, L20, L30), either cold-stored (L1C4, L2C4, L3C3, L1C7, L2C7 and L3C6), or cold-stored N_2_-flushed (L1N4, L2N4, L3N3, L1N7, L2N7 and L3N6) raw milk. Phylogenetic position is based on the results of the phylogenetic analysis shown in S1 Fig.

RELATIVE ABUNDANCE (%)																	
OTU no	Phylogenetic position	L10	L20	L30	L1C4	L2C4	L3C3	L1N4	L2N4	L3N3	L1C7	L2C7	L3C6	L1N7	L2N7	L3N6	Occurrence
**0001**	*P*. *veronii*	97	80	62	94	67	92	93	71	79	94	61	82	77	64	81	15/15
**0031**	*P*. *nitroreducens*	0	0	32	0	29	6	0	26	18	1	36	15	0	0	16	9/15
**0090**	*P*. *lundensis*	0	0	3	2	2	0	2	2	0	3	2	0	3	3	0	9/15
**0061**	*P*. *fragi*	0	0	0	0	0	1	0	0	0	0	0	2	6	1	1	5/15
**0002**	*P*. *nitroreducens*	1	0	0	2	0	0	2	0	0	0	0	0	13	0	0	4/15
**0004**	*P*. *alcaligenes*	0	17	0	0	0	0	0	0	1	0	0	0	0	29	0	3/15
**0062**	*P*. *putida*	0	2	0	0	0	0	0	0	0	0	0	0	0	0	0	1/15
**0010**	*P*. *lini*	0	0	0	0	0	0	1	0	0	0	0	0	0	0	0	1/15
**0430**	*P*. *panipatensis*	1	0	0	0	0	0	0	0	0	0	0	0	0	0	0	1/15
**Others**	*Pseudomonas*	1	1	3	2	2	1	2	1	2	2	1	1	1	3	2	-

Other genera of Proteobacteria harboring strains with pathogenic potential (*Yersinia*, *Serratia*, *Hafnia*, *Delftia*, *Campylobacter*, *Escherichia-Shigella)* accounted for only 2.0–7.2% of all reads in total (Table 2, S1 Table). Overall, no bacteria associated with spoilage or human pathogenic features, or anaerobes were clearly stimulated by the N_2_-gas-flushing treatment (Table 2, S1 Table).

Rare taxa (OTUs <1%) were present in all samples, irrespective the conditions or sampling times. They made up over 20% of the OTUs for the initial raw-milk samples (data not shown). Over time, and irrespective of the storage conditions, the level of rare taxa dropped in relative amounts to 5–10% in the milk samples subjected to cold storage alone. In contrast, the cold-stored N_2_-flushed samples contained with one exception (L2N4), the less frequent OTUs (<1%) in higher abundance (approximately 20%) compared to their corresponding controls, and equivalent to their initial levels (L10, L20 and L30) (S1 Table).

## Discussion

Raw milk contains microbes, mainly bacteria, which may be technologically relevant or associated with spoilage or with human health [22]. The initial raw-milk samples considered in this study were of very good microbiological quality, exhibiting initial counts of approximately 10^4^ cfu/ml [1]; the culture-based analyses indicated that bacterial growth in raw milk was inhibited at 6°C by N_2_ gas-flushing (Fig 1), as previously observed [12].

Our data, obtained from 16S rRNA-based barcoding of bacteria, confirmed results from earlier studies that showed that cold storage of milk triggers changes in bacterial populations, whether evaluated by cultivation-dependent [3, 6] or cultivation-independent studies [9, 23]. Flushing with N_2_ gas clearly selected for different bacterial phylotypes compared to the corresponding controls (Figs 4 and 5, Tables 1 and 2, S1 Table). Indeed, the tendency towards a decreased OTU richness under cold storage was also observed for the N_2_-flushed milk samples; but overall bacterial diversity declined to a lesser degree, in samples that were treated with a combination of cold storage and N_2_ flushing, compared to the cold storage alone (Figs 3–4). Primarily, rare taxa were better preserved by N_2_ gas flushing compared to the cold storage alone.

Considering that the initial raw-milk samples had been in cold storage for up to two days in various farm tanks prior to collection and that the three raw-milk samples represent commingled milk produced in different months, the results for the bacterial diversity pattern in the initial three raw- milk samples in terms of relative abundance of the major groups are very consistent (S1 Table). Initial raw-milk samples were dominated by rRNA sequences related to Firmicutes, which is consistent with previous studies [24, 25], even though the relative abundance of Firmicutes was lower in this study. However, a large number of reads could not be clearly assigned to particular genera in this group, as the amplified V1 –V2 region of the 16S rRNA gene had only low resolution for this group.

For the initial raw-milk samples, our study revealed that approximately 1% of the reads could be assigned to the genus *Jeotgalicoccus* (Fig 5, Table 2, S1 Table). *J*. *psychrophilus*, which grows between 4 to 34°C, was found in the teat canal of cow udders, and was considered to be an atypical halophilic species in goat´s milk. [26–28]. Reads related to bacteria of the genus *Oscillibacter* were also found in the initial raw-milk samples (Figs 4H and 5, Table 2, S1 Table). *Oscillibacter* are strictly anaerobic, mesophilic, nonsporeforming bacteria that have been so far primarily described as members of the swine gut microbiome [29, 30]. Recently, cases of bacteremia caused by *O*. *ruminatium* have been reported [31]. The presence of *Oscillibacter*-like sequences was also reported in human-milk samples [32]. Overall, neither the genus *Oscillibacter* nor *Jeotgalicoccus* were detected in raw-milk samples that were stored for 6 to 7 days, irrespective of the treatment (Fig 5). Bacteria belonging to Bacteroidetes were also negatively impacted by both storage conditions tested (Fig 4A, S1 Table). In this study, bacteria of the genera *Alistipes* and *Chryseobacterium* predominated in the initial raw-milk samples (S1 Table). *Alistipes* bacteria were detected in cattle feces [33] and bacteria belonging to *Chryseobacterium* were previously isolated from bovine milk [34–36]. Major gut-associated obligate anaerobes such as *Bacteroidetes* were also found in breast milk [37]. Our study revealed that some taxa, especially bacteria belonging to Firmicutes such as *Jeotgalicoccus*, *Oscillibacter*, Lachnospiraceae, Clostridiales and also to Bacteroidetes (Bacteroidales) were particularly sensitive to cold storage (Fig 5). This characteristic could be used as an indicator of the “freshness” of cold-stored raw milk.

Storage at 6°C increased the number of reads for proteobacteria mainly of the genera *Pseudomonas* and *Acinetobacter*, suggesting that the major psychrotrophs belong to these bacterial groups (Figs 4J and 5, Table 2, S1 Table). This agrees with many reports concerning psychrotrophs in raw milk [3, 4, 5, 8, 10, 38, 39]. A previous study suggested that among Proteobacteria, *Acinetobacter* was more abundant during early cold storage, whereas *Pseudomonas* was predominant during the late cold storage phase [10]. Similarly, in this study, *Acinetobacter*-specific rRNA increased after 3 to 4 days of cold storage and remained stable thereafter, while *Pseudomonas*-specific rRNA was highest after 6 to 7 days (Figs 4 and 5, Table 2, S1 Table). Both groups, especially *Pseudomonas*, were negatively affected by additional N_2_ flushing, and the number of reads was significantly lower at both sampling times compared to samples that had only been cold-stored (Fig 5, S1 Table).

That N_2_ flushing has a negative impact on *Acinetobacter* is important because members of this genus are known to harbor multiple antibiotic-resistance genes; multi-resistant isolates of *Acinetobacter* were also found in raw milk [40].

The genus *Pseudomonas* is very heterogeneous and ubiquitous in nature; it comprises many psychrotrophs which have the remarkable ability to produce heat-resistant enzymes (protease, lipase, phospholipase) that can alter or spoil food products [2–8, 41, 42]. The diversity of pseudomonads in raw milk is also illustrated in this study as the group contained the largest number of OTUs (257) (Table 2); however, 248 of these OTUs were affiliated with taxa present at levels below 1%; therefore, rare members of the microbial community constitute the vast majority of the diversity encountered in the genus *Pseudomonas*.

The nine dominating pseudomonad OTUs, present at levels above 1%, were affiliated with *P*. *veronii*, *P*. *nitroreducens*, *P*. *alcaligenes*, *P*. *fragi*, *P*. *putida*, *P*. *lundensis*, and *P*. *panipatensis* (Table 3, S1 Fig). Although some species such as *P*. *fragi*, *P*. *putida* or *P*. *lundensis* are common raw-milk inhabitants, because they were found to be associated with milk spoilage with culture-dependent or culture-independent methods [4, 39, 43, 44], the absence of *P*. *fluorescens* was surprising considering the multitude of reports that described the psychrotolerant *P*. *fluorescens* as the key species responsible for milk spoilage with consequences on various dairy products. *P*. *putida*, also known for its versatile metabolic ability, is reported to be commonly found in raw milk and is also reputed to be a common milk spoiler along with *P*. *fluorescens* [4, 7, 39].

Surprisingly, a single OTU that was phylogenetically most closely related to *P*. *veronii* and ranged in relative abundance from 60 over 97%, was found to be widely distributed in all of the samples (Table 3). The species, originally isolated from natural mineral water, is lipase- and phospholipase- negative, produces a fluorescent pigment on KB medium, is capable of growth at 4°C, and exhibits physiological flexibility during periods of anoxia [45, 46]. Several reports highlighted the crucial role of water as a major source of pseudomonal contamination of milk [5, 47–49]. All preceding observations could explain why OTU 0001 was equally dominant in the initial stages of the analyses but also in cold-stored milk where the O_2_ tension is lower because of excessive bacterial growth, or in N_2_-flushed milk where O_2_ is absent. Culture-based studies and the inherent limitations of phenotypic identification systems, which were widely applied to investigate earlier raw-milk-spoiling *Pseudomonas*, could explain discrepancies with the present study.

Overall, it must be taken into account that our study was based on rRNA analysis and not on that of DNA, as in former studies [22, 24, 37, 50–57], which might have induced differences. Thus in our study, mainly bacteria with a high rRNA content (as a result of high activity) were targeted [15]. In contrast, the DNA content of the cells is more uniform, although differences in the numbers of rRNA operons have been reported [58, 59]. Furthermore, RNA is quickly degraded during cellular decay, but DNA can remain stable for several weeks or months [60], depending on the matrix. Bacterial DNA, present after cell death, may lead to erroneous conclusions if bacteria are targeted by a DNA-based barcoding approach. However, the greatest bias introduced during bacterial barcoding originates from the nucleic acid extraction procedure (mainly from the cell lysis procedure) and the subsequent PCR, as all primers, assumed to be universal, introduce bias to an unknown extent [61–63]. Finally, the sample storage conditions prior to nucleic acid extraction strongly interfere with the barcodes obtained; for example, sample freeze-thaw procedures might induce a large shift in the bacterial community structure, as has been shown in fecal samples [64]; this point could perhaps explain why strictly anaerobic bacterial groups such as Bacteroidales, Ruminococcaceae, Clostridiales, Lachnospiraceaea found in this study were practically absent in two previous studies on bovine raw milk [24, 25].

## Conclusion

Our data indicate that bacterial diversity is better preserved in bovine raw milk by additional flushing with N_2_ gas compared to cold storage at 6°C alone. Most interestingly, the study revealed that no well-known human pathogens, milk spoilers, or anaerobes were clearly favored by the N_2_ -flushing treatment, indicating a higher potential for N_2_ gas flushing as a supplementary treatment to preserve the quality and safety of raw milk during the raw-milk cold-storage and transportation chain.

As suspected from earlier observations, based on culture–dependent methods, this study confirmed that N_2_ gas flushing particularly affected *Pseudomonas*. However, the type of storage did not change the diversity pattern of this genus. In all samples, OTUs related to *Pseudomonas veronii* species were predominant. Some groups of anaerobes, mostly typical intestinal bacteria, were also major bacterial groups found in initial raw-milk samples; however, they were sensitive to cold- storage conditions.

These data are highly promising as they might enable dairy manufacturers to achieve a higher bacterial diversity in their products. Furthermore, the results presented herein might allow for the extension of the cold-storage time of raw milk without negative impact on microbiological quality.

## Supporting Information

S1 Fig Phylogenetic dendrogram (maximum likelihood consensus tree) showing the distribution of OTUs (>4 reads) related to *Pseudomonas* derived from initial raw-milk samples, and from cold- stored raw milk with or without N_2_ flushing for 3 to 4 and 6 to 7 days. The OTUs above 1% of relative abundance are highlighted in grey.
(PDF)

S1 Table Relative abundance of bacterial OTUs (97% similarity) from initial raw-milk samples, and from cold-stored milks for 3 to 4 or 6 to 7 days with or without N_2_ gas flushing. (DOCX)
