# Supplementary figures and images for: N2 Gas Flushing Alleviates the Loss of Bacterial Diversity and Inhibits Psychrotrophic Pseudomonas during the Cold Storage of Bovine Raw Milk
Source: PLoS One. 2016 Jan 5;11(1):e0146015. doi: 10.1371/journal.pone.0146015 (PMC4701220; doi:10.1371/journal.pone.0146015)

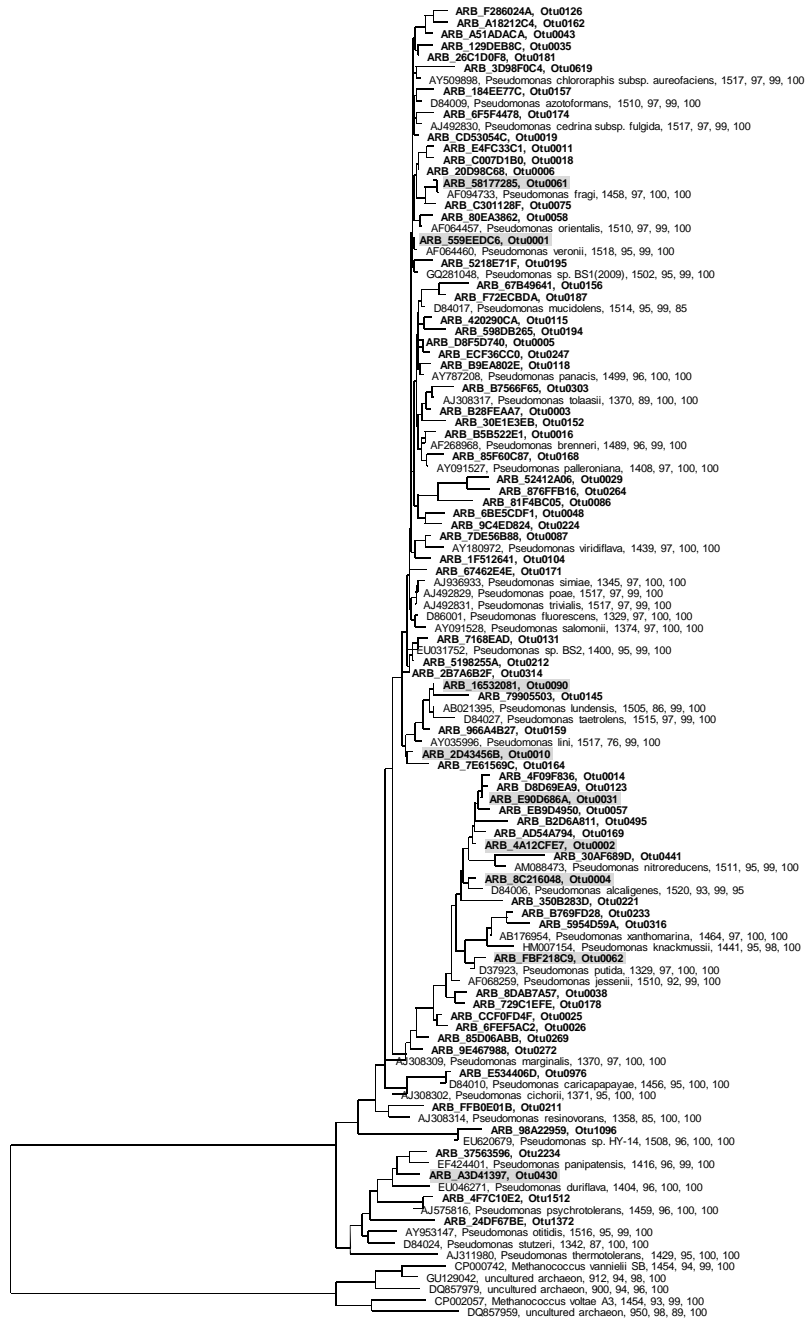

Supplement: S1 Fig — The OTUs above 1% of relative abundance are highlighted in grey. (PDF) [file pone.0146015.s001.pdf]
