# Supplementary material for: N2 Gas Flushing Alleviates the Loss of Bacterial Diversity and Inhibits Psychrotrophic Pseudomonas during the Cold Storage of Bovine Raw Milk
Source: PLoS One. 2016 Jan 5;11(1):e0146015. doi: 10.1371/journal.pone.0146015 (PMC4701220; doi:10.1371/journal.pone.0146015)
Supplement: S1 Table — (DOCX) [file pone.0146015.s002.docx]

S1 Table. Relative abundance (%) of all bacterial taxa observed in initial (L10, L20, L30) and either cold stored (L1C4, L2C4, L3C3, L1C7, L2C7, L3C6) or cold stored while N_2_ flushed (L1N4, L2N4, L3N3, L1N7, L2N7, L3N6) raw milks.

|  |  |  |  |  |  |  |  |  |  |  |  |  |  |  |  |  |  |  |  |  |
| --- | --- | --- | --- | --- | --- | --- | --- | --- | --- | --- | --- | --- | --- | --- | --- | --- | --- | --- | --- | --- |
| Taxon |  | Initial |  |  |  | Cold stored |  |  |  | Cold stored  +N_2_ flushed |  |  |  | Cold stored |  |  |  | Cold stored  +N_2_ flushed |  |  |
|  |  | 0 day | 0 day | 0 day |  | 4 days | 4 days | 3 days |  | 4 days | 4 days | 3 days |  | 7 days | 7 days | 6 days |  | 7 days | 7 days | 6 days |
|  |  | L10 | L20 | L30 |  | L1C4 | L2C4 | L3C3 |  | L1N4 | L2N4 | L3N3 |  | L1C7 | L2C7 | L3C6 |  | L1N7 | L2N7 | L3N6 |
| Phylum | Genus ^a^ | 43427* | 44082 | 46567 |  | 47145 | 48167 | 60252 |  | 49706 | 64464 | 41980 |  | 47976 | 56069 | 66224 |  | 50406 | 34356 | 72317 |
| *Acidobacteria* | *Gp16* | 0.0 | 0.1 | 0.0 |  | 0.0 | 0.0 | 0.0 |  | 0.0 | 0.0 | 0.0 |  | 0.0 | 0.0 | 0.0 |  | 0.0 | 0.0 | 0.0 |
|  | *Gp3* | 0.2 | 0.0 | 0.0 |  | 0.0 | 0.0 | 0.0 |  | 0.0 | 0.0 | 0.0 |  | 0.0 | 0.0 | 0.0 |  | 0.0 | 0.0 | 0.0 |
|  | *Gp4* | 0.0 | 0.0 | 0.0 |  | 0.0 | 0.0 | 0.0 |  | 0.6 | 0.0 | 0.0 |  | 0.0 | 0.0 | 0.0 |  | 0.0 | 0.0 | 0.0 |
|  | *Gp6* | 0.2 | 0.4 | 0.0 |  | 0.0 | 0.0 | 0.0 |  | 1.0 | 0.0 | 0.3 |  | 0.0 | 0.0 | 0.0 |  | 0.3 | 0.0 | 0.0 |
| *Actinobacteria* | *Ilumatobacter* | 0.4 | 0.2 | 0.0 |  | 0.0 | 0.5 | 0.0 |  | 0.6 | 0.0 | 0.0 |  | 0.0 | 1.0 | 0.0 |  | 0.6 | 1.0 | 0.0 |
|  | *Iamia* | 0.2 | 0.1 | 0.0 |  | 0.0 | 0.0 | 0.0 |  | 0.0 | 0.0 | 0.0 |  | 0.0 | 0.0 | 0.0 |  | 0.0 | 0.0 | 0.0 |
|  | *Acidimicrobiales_u* | 0.2 | 0.0 | 0.0 |  | 0.0 | 0.0 | 0.0 |  | 0.0 | 0.0 | 0.1 |  | 0.0 | 0.0 | 0.0 |  | 0.0 | 0.3 | 0.0 |
|  | *Actinomyces* | 0.2 | 0.4 | 0.2 |  | 0.0 | 0.0 | 0.6 |  | 0.6 | 0.0 | 0.6 |  | 0.0 | 0.0 | 0.0 |  | 0.0 | 0.3 | 0.0 |
|  | *Actinomycetaceae_u* | 0.4 | 0.4 | 0.0 |  | 0.0 | 0.0 | 0.0 |  | 0.0 | 0.0 | 0.0 |  | 0.0 | 0.0 | 0.0 |  | 0.0 | 0.0 | 0.4 |
|  | *Brevibacterium* | 1.6 | 1.0 | 0.4 |  | 1.2 | 0.5 | 1.8 |  | 2.3 | 0.6 | 0.8 |  | 0.6 | 1.0 | 1.7 |  | 0.9 | 1.0 | 1.1 |
|  | *Corynebacterium* | 2.5 | 2.6 | 2.7 |  | 1.5 | 0.9 | 2.9 |  | 2.6 | 0.6 | 3.1 |  | 0.6 | 1.0 | 1.7 |  | 1.2 | 1.7 | 2.3 |
|  | *Corynebacteriaceae_u* | 0.0 | 0.1 | 0.0 |  | 0.0 | 0.0 | 0.0 |  | 0.0 | 0.0 | 0.0 |  | 0.0 | 0.0 | 0.0 |  | 0.0 | 0.0 | 0.0 |
|  | *Brachybacterium* | 0.4 | 0.2 | 0.4 |  | 0.8 | 0.5 | 1.2 |  | 0.6 | 0.6 | 0.1 |  | 1.1 | 0.5 | 0.8 |  | 0.9 | 0.7 | 0.8 |
|  | *Dermabacter* | 0.0 | 0.0 | 0.0 |  | 0.0 | 0.0 | 0.0 |  | 0.0 | 0.0 | 0.1 |  | 0.0 | 0.0 | 0.0 |  | 0.0 | 0.0 | 0.0 |
|  | *Dermacoccus* | 0.0 | 0.0 | 0.0 |  | 0.0 | 0.0 | 0.0 |  | 0.0 | 0.0 | 0.0 |  | 0.6 | 0.0 | 0.0 |  | 0.3 | 0.0 | 0.0 |
|  | *Piscicoccus* | 0.0 | 0.0 | 0.0 |  | 0.0 | 0.0 | 0.0 |  | 0.0 | 0.0 | 0.1 |  | 0.0 | 0.0 | 0.0 |  | 0.0 | 0.0 | 0.0 |
|  | *Dermatophilaceae_u* | 0.0 | 0.0 | 0.2 |  | 0.0 | 0.0 | 0.0 |  | 0.0 | 0.0 | 0.0 |  | 0.0 | 0.0 | 0.0 |  | 0.0 | 0.0 | 0.0 |
|  | *Dietzia* | 0.2 | 0.2 | 0.0 |  | 0.0 | 0.0 | 0.0 |  | 0.0 | 0.0 | 0.1 |  | 0.0 | 0.0 | 0.0 |  | 0.0 | 0.0 | 0.0 |
|  | *Blastococcus* | 0.2 | 0.2 | 0.0 |  | 0.0 | 0.0 | 0.0 |  | 0.0 | 0.0 | 0.1 |  | 0.0 | 0.0 | 0.0 |  | 0.3 | 0.0 | 0.0 |
|  | *Modestobacter* | 0.2 | 0.0 | 0.0 |  | 0.0 | 0.0 | 0.0 |  | 0.3 | 0.0 | 0.0 |  | 0.0 | 0.0 | 0.0 |  | 0.3 | 0.0 | 0.0 |
|  | *Janibacter* | 0.2 | 0.0 | 0.0 |  | 0.0 | 0.0 | 0.0 |  | 0.0 | 0.0 | 0.0 |  | 0.0 | 0.0 | 0.0 |  | 0.0 | 0.0 | 0.0 |
|  | *Knoellia* | 0.0 | 0.0 | 0.0 |  | 0.0 | 0.0 | 0.0 |  | 0.0 | 0.0 | 0.0 |  | 0.0 | 0.0 | 0.0 |  | 0.3 | 0.0 | 0.0 |
|  | *Ornithinibacter* | 0.0 | 0.1 | 0.0 |  | 0.0 | 0.0 | 0.0 |  | 0.0 | 0.0 | 0.0 |  | 0.0 | 0.0 | 0.0 |  | 0.0 | 0.0 | 0.0 |
|  | *Ornithinimicrobium* | 0.4 | 0.1 | 0.0 |  | 0.0 | 0.0 | 0.0 |  | 0.0 | 0.0 | 0.0 |  | 0.0 | 0.0 | 0.0 |  | 0.0 | 0.0 | 0.0 |
|  | *Terracoccus* | 0.0 | 0.1 | 0.0 |  | 0.0 | 0.0 | 0.0 |  | 0.0 | 0.0 | 0.0 |  | 0.0 | 0.0 | 0.0 |  | 0.0 | 0.0 | 0.0 |
|  | *Tetrasphaera* | 0.2 | 0.1 | 0.0 |  | 0.0 | 0.0 | 0.0 |  | 0.3 | 0.6 | 0.0 |  | 0.0 | 0.0 | 0.0 |  | 0.0 | 0.0 | 0.0 |
|  | *Intrasporangiaceae_u* | 0.4 | 0.1 | 0.0 |  | 0.0 | 0.0 | 0.0 |  | 0.0 | 0.0 | 0.0 |  | 0.0 | 0.0 | 0.0 |  | 0.0 | 0.0 | 0.0 |
|  | *Angustibacter* | 0.0 | 0.0 | 0.0 |  | 0.0 | 0.0 | 0.0 |  | 0.0 | 0.0 | 0.1 |  | 0.0 | 0.0 | 0.0 |  | 0.0 | 0.0 | 0.0 |
|  | *Kineococcus* | 0.0 | 0.0 | 0.0 |  | 0.0 | 0.0 | 0.0 |  | 0.3 | 0.0 | 0.1 |  | 0.0 | 0.5 | 0.0 |  | 0.0 | 0.0 | 0.0 |
|  | *Agrococcus* | 0.2 | 0.0 | 0.0 |  | 0.0 | 0.0 | 0.0 |  | 0.0 | 0.0 | 0.0 |  | 0.0 | 0.0 | 0.0 |  | 0.0 | 0.0 | 0.0 |
|  | *Clavibacter* | 0.0 | 0.0 | 0.2 |  | 0.0 | 0.0 | 0.0 |  | 0.0 | 0.0 | 0.0 |  | 0.0 | 0.0 | 0.0 |  | 0.0 | 0.0 | 0.0 |
|  | *Leucobacter* | 0.0 | 0.0 | 0.0 |  | 0.4 | 0.0 | 0.0 |  | 0.0 | 0.0 | 0.0 |  | 0.0 | 0.0 | 0.0 |  | 0.0 | 0.0 | 0.0 |
|  | *Microbacterium* | 0.5 | 0.4 | 0.4 |  | 0.4 | 0.5 | 0.0 |  | 1.6 | 0.0 | 0.4 |  | 0.0 | 0.0 | 0.0 |  | 0.3 | 1.0 | 0.4 |
|  | *Okibacterium* | 0.0 | 0.1 | 0.0 |  | 0.0 | 0.0 | 0.0 |  | 0.0 | 0.0 | 0.0 |  | 0.0 | 0.0 | 0.0 |  | 0.0 | 0.0 | 0.0 |
|  | *Pseudoclavibacter* | 0.0 | 0.0 | 0.0 |  | 0.0 | 0.5 | 0.0 |  | 0.0 | 0.0 | 0.0 |  | 0.0 | 0.0 | 0.0 |  | 0.0 | 0.3 | 0.0 |
|  | *Rathayibacter* | 0.0 | 0.0 | 0.0 |  | 0.0 | 0.0 | 0.0 |  | 0.0 | 0.0 | 0.0 |  | 0.0 | 0.0 | 0.0 |  | 0.3 | 0.0 | 0.0 |
|  | *Zimmermannella* | 0.2 | 0.0 | 0.4 |  | 0.0 | 0.0 | 0.0 |  | 0.0 | 0.0 | 0.0 |  | 0.0 | 0.0 | 0.0 |  | 0.0 | 0.0 | 0.0 |
|  | *Microbacteriaceae_u* | 0.0 | 0.2 | 0.0 |  | 0.0 | 0.0 | 0.0 |  | 0.3 | 0.0 | 0.0 |  | 0.0 | 0.0 | 0.0 |  | 0.0 | 0.0 | 0.0 |
|  | *Arthrobacter* | 0.4 | 0.2 | 0.0 |  | 0.0 | 0.5 | 0.0 |  | 0.3 | 0.0 | 0.0 |  | 0.0 | 0.0 | 0.0 |  | 0.3 | 0.3 | 0.4 |
|  | *Citricoccus* | 0.0 | 0.1 | 0.0 |  | 0.0 | 0.0 | 0.0 |  | 0.0 | 0.0 | 0.0 |  | 0.0 | 0.0 | 0.0 |  | 0.0 | 0.0 | 0.0 |
|  | *Kocuria* | 2.3 | 0.6 | 0.4 |  | 1.5 | 0.9 | 1.8 |  | 1.9 | 1.1 | 1.1 |  | 0.6 | 1.4 | 0.8 |  | 0.9 | 0.7 | 1.5 |
|  | *Micrococcus* | 0.7 | 0.4 | 0.4 |  | 0.8 | 0.9 | 2.3 |  | 1.0 | 1.1 | 0.6 |  | 0.6 | 0.5 | 0.8 |  | 0.9 | 0.7 | 1.1 |
|  | *Rothia* | 0.2 | 0.0 | 0.0 |  | 0.0 | 0.0 | 0.6 |  | 0.3 | 0.0 | 0.4 |  | 0.0 | 0.0 | 0.8 |  | 0.6 | 0.0 | 0.4 |
|  | *Micrococcaceae_u* | 0.4 | 0.1 | 0.0 |  | 0.0 | 0.0 | 0.0 |  | 0.3 | 0.0 | 0.3 |  | 0.0 | 0.5 | 0.0 |  | 0.3 | 0.3 | 0.0 |
|  | *Actinoplanes* | 0.0 | 0.1 | 0.0 |  | 0.0 | 0.0 | 0.0 |  | 0.0 | 0.0 | 0.0 |  | 0.0 | 0.0 | 0.0 |  | 0.0 | 0.0 | 0.0 |
|  | *Micromonosporaceae_u* | 0.0 | 0.0 | 0.0 |  | 0.0 | 0.0 | 0.0 |  | 0.0 | 0.0 | 0.0 |  | 0.0 | 0.0 | 0.0 |  | 0.0 | 0.3 | 0.0 |
|  | *Mycobacterium* | 0.0 | 0.1 | 0.0 |  | 0.4 | 0.0 | 0.0 |  | 0.0 | 0.0 | 0.0 |  | 0.0 | 0.0 | 0.0 |  | 0.3 | 0.3 | 0.0 |
|  | *Humicoccus* | 0.0 | 0.0 | 0.0 |  | 0.0 | 0.5 | 0.0 |  | 0.6 | 0.0 | 0.1 |  | 0.0 | 0.0 | 0.0 |  | 0.3 | 0.0 | 0.0 |
|  | *Nakamurellaceae_u* | 0.2 | 0.1 | 0.0 |  | 0.0 | 0.0 | 0.0 |  | 0.0 | 0.0 | 0.0 |  | 0.6 | 0.0 | 0.0 |  | 0.3 | 0.0 | 0.0 |
|  | *Gordonia* | 0.2 | 0.1 | 0.0 |  | 0.0 | 0.0 | 0.0 |  | 0.0 | 0.0 | 0.1 |  | 0.0 | 0.0 | 0.0 |  | 0.0 | 0.0 | 0.0 |
|  | *Rhodococcus* | 0.0 | 0.1 | 0.0 |  | 0.0 | 0.0 | 0.0 |  | 0.0 | 0.0 | 0.0 |  | 0.0 | 0.0 | 0.0 |  | 0.0 | 0.3 | 0.0 |
|  | *Marmoricola* | 0.4 | 0.1 | 0.0 |  | 0.0 | 0.5 | 0.0 |  | 0.3 | 0.0 | 0.0 |  | 0.0 | 0.0 | 0.8 |  | 0.3 | 0.7 | 0.0 |
|  | *Nocardioides* | 0.5 | 0.6 | 0.0 |  | 0.0 | 0.5 | 0.0 |  | 1.0 | 0.6 | 0.0 |  | 0.0 | 0.0 | 0.0 |  | 0.3 | 1.0 | 0.0 |
|  | *Nocardioidaceae_u* | 0.0 | 0.2 | 0.0 |  | 0.4 | 0.0 | 0.0 |  | 0.0 | 0.0 | 0.0 |  | 0.0 | 0.0 | 0.0 |  | 0.0 | 0.0 | 0.0 |
|  | *Friedmanniella* | 0.2 | 0.1 | 0.0 |  | 0.0 | 0.0 | 0.0 |  | 0.3 | 0.0 | 0.1 |  | 0.0 | 0.0 | 0.0 |  | 0.6 | 0.0 | 0.0 |
|  | *Luteococcus* | 0.5 | 0.1 | 0.0 |  | 0.0 | 0.0 | 0.0 |  | 0.0 | 0.0 | 0.0 |  | 0.0 | 0.0 | 0.0 |  | 0.0 | 0.0 | 0.0 |
|  | *Microlunatus* | 0.2 | 0.2 | 0.0 |  | 0.0 | 0.0 | 0.0 |  | 0.0 | 0.0 | 0.0 |  | 0.0 | 0.0 | 0.0 |  | 0.0 | 0.0 | 0.0 |
|  | *Propionibacterium* | 0.5 | 0.2 | 0.4 |  | 0.4 | 0.5 | 1.2 |  | 0.3 | 0.6 | 0.4 |  | 0.6 | 1.0 | 0.0 |  | 0.6 | 0.7 | 0.4 |
|  | *Tessaracoccus* | 0.0 | 0.0 | 0.2 |  | 0.0 | 0.0 | 0.0 |  | 0.0 | 0.0 | 0.0 |  | 0.0 | 0.0 | 0.0 |  | 0.0 | 0.0 | 0.0 |
|  | *Propionibacteriaceae_u* | 0.2 | 0.4 | 0.2 |  | 0.0 | 0.0 | 0.0 |  | 0.0 | 0.0 | 0.0 |  | 0.0 | 0.0 | 0.0 |  | 0.0 | 0.0 | 0.0 |
|  | *Actinomycetospora* | 0.2 | 0.2 | 0.0 |  | 0.0 | 0.0 | 0.0 |  | 0.0 | 0.0 | 0.0 |  | 0.0 | 0.0 | 0.0 |  | 0.0 | 0.0 | 0.0 |
|  | *Pseudonocardia* | 0.2 | 0.1 | 0.0 |  | 0.0 | 0.0 | 0.0 |  | 0.3 | 0.0 | 0.0 |  | 0.0 | 0.0 | 0.0 |  | 0.0 | 0.3 | 0.0 |
|  | *Sanguibacter* | 0.2 | 0.0 | 0.2 |  | 0.0 | 0.0 | 0.0 |  | 0.3 | 0.6 | 0.0 |  | 0.6 | 0.0 | 0.0 |  | 0.3 | 0.0 | 0.0 |
|  | *Actinomycetales_u* | 1.2 | 0.7 | 0.2 |  | 0.4 | 0.0 | 1.2 |  | 1.6 | 0.0 | 0.6 |  | 0.0 | 0.0 | 0.0 |  | 0.9 | 0.7 | 0.0 |
|  | *Atopobium* | 0.0 | 0.0 | 0.0 |  | 0.0 | 0.0 | 0.0 |  | 0.0 | 0.0 | 0.0 |  | 0.0 | 0.0 | 0.0 |  | 0.0 | 0.0 | 0.4 |
|  | *Olsenella* | 0.0 | 0.0 | 0.0 |  | 0.0 | 0.0 | 0.0 |  | 0.0 | 0.0 | 0.0 |  | 0.0 | 0.0 | 0.8 |  | 0.0 | 0.0 | 0.0 |
|  | *Coriobacteriaceae_u* | 0.0 | 0.0 | 0.0 |  | 0.0 | 0.0 | 0.0 |  | 0.0 | 0.6 | 0.1 |  | 0.0 | 0.0 | 0.0 |  | 0.0 | 0.0 | 0.0 |
|  | *Patulibacter* | 0.2 | 0.1 | 0.0 |  | 0.0 | 0.0 | 0.0 |  | 0.0 | 0.0 | 0.0 |  | 0.0 | 0.0 | 0.0 |  | 0.3 | 0.0 | 0.0 |
|  | *Solirubrobacter* | 0.2 | 0.2 | 0.2 |  | 0.0 | 0.0 | 0.0 |  | 0.0 | 0.0 | 0.0 |  | 0.0 | 0.0 | 0.0 |  | 0.0 | 0.3 | 0.0 |
|  | *Solirubrobacterales_u* | 0.2 | 0.1 | 0.2 |  | 0.0 | 0.0 | 0.0 |  | 0.6 | 0.0 | 0.3 |  | 0.0 | 0.0 | 0.0 |  | 0.0 | 0.0 | 0.0 |
|  | *Actinobacteria_u* | 0.2 | 0.2 | 0.0 |  | 0.0 | 0.0 | 0.0 |  | 0.0 | 0.0 | 0.0 |  | 0.0 | 0.0 | 0.0 |  | 0.0 | 0.0 | 0.0 |
| *Armatimonadetes* | *Armatimonas_Gp1* | 0.0 | 0.1 | 0.0 |  | 0.0 | 0.0 | 0.0 |  | 0.0 | 0.0 | 0.0 |  | 0.0 | 0.0 | 0.0 |  | 0.0 | 0.0 | 0.0 |
| *Bacteroidetes* | *Dysgonomonas* | 0.0 | 0.1 | 0.2 |  | 0.0 | 0.0 | 0.0 |  | 0.0 | 0.0 | 0.1 |  | 0.0 | 0.0 | 0.0 |  | 0.0 | 0.0 | 0.0 |
|  | *Odoribacter* | 0.0 | 0.1 | 0.0 |  | 0.0 | 0.0 | 0.0 |  | 0.0 | 0.0 | 0.0 |  | 0.0 | 0.0 | 0.0 |  | 0.0 | 0.0 | 0.0 |
|  | *Paludibacter* | 0.2 | 0.1 | 0.2 |  | 0.0 | 0.0 | 0.0 |  | 0.0 | 0.0 | 0.1 |  | 0.0 | 0.0 | 0.0 |  | 0.0 | 0.0 | 0.4 |
|  | *Parabacteroides* | 0.0 | 0.0 | 0.0 |  | 0.0 | 0.0 | 0.0 |  | 0.0 | 0.0 | 0.3 |  | 0.0 | 0.0 | 0.0 |  | 0.0 | 0.0 | 0.0 |
|  | *Porphyromonas* | 0.2 | 0.2 | 0.6 |  | 0.0 | 0.0 | 0.0 |  | 0.0 | 0.0 | 0.0 |  | 0.0 | 0.0 | 0.0 |  | 0.3 | 0.0 | 0.0 |
|  | *Proteiniphilum* | 0.0 | 0.0 | 0.4 |  | 0.0 | 0.0 | 0.0 |  | 0.0 | 0.0 | 0.0 |  | 0.0 | 0.0 | 0.0 |  | 0.0 | 0.0 | 0.0 |
|  | *Porphyromonadaceae_u* | 0.2 | 0.7 | 3.3 |  | 0.0 | 0.0 | 0.0 |  | 0.0 | 0.0 | 1.1 |  | 0.0 | 0.0 | 0.0 |  | 0.0 | 0.0 | 0.8 |
|  | *Paraprevotella* | 0.2 | 0.0 | 0.0 |  | 0.0 | 0.0 | 0.0 |  | 0.0 | 0.0 | 0.3 |  | 0.0 | 0.0 | 0.0 |  | 0.0 | 0.0 | 0.0 |
|  | *Prevotella* | 0.5 | 0.0 | 0.2 |  | 0.4 | 0.0 | 0.0 |  | 0.0 | 0.0 | 0.1 |  | 0.0 | 0.0 | 0.0 |  | 0.0 | 0.0 | 0.0 |
|  | *Prevotellaceae_u* | 0.4 | 0.4 | 1.0 |  | 0.0 | 0.0 | 1.2 |  | 0.0 | 0.6 | 0.7 |  | 0.0 | 0.0 | 0.0 |  | 0.0 | 0.0 | 0.0 |
|  | *Alistipes* | 1.4 | 3.2 | 2.3 |  | 0.0 | 0.0 | 1.2 |  | 0.3 | 0.0 | 2.8 |  | 0.0 | 0.0 | 0.0 |  | 0.3 | 0.0 | 1.1 |
|  | *Bacteroidales_u* | 5.2 | 4.9 | 4.7 |  | 0.8 | 0.5 | 2.3 |  | 1.0 | 0.0 | 4.3 |  | 0.6 | 0.0 | 0.0 |  | 0.9 | 0.3 | 0.8 |
|  | *Flavisolibacter* | 0.2 | 0.0 | 0.0 |  | 0.0 | 0.0 | 0.0 |  | 0.0 | 0.0 | 0.0 |  | 0.0 | 0.0 | 0.0 |  | 0.0 | 0.0 | 0.0 |
|  | *Chitinophagaceae_u* | 0.4 | 0.6 | 0.0 |  | 0.0 | 0.0 | 0.0 |  | 1.0 | 0.0 | 0.1 |  | 0.0 | 0.0 | 0.0 |  | 0.3 | 0.3 | 0.0 |
|  | *Hymenobacter* | 0.0 | 0.5 | 0.2 |  | 0.0 | 0.0 | 0.0 |  | 0.3 | 0.0 | 0.0 |  | 0.0 | 0.0 | 0.0 |  | 0.0 | 0.3 | 0.0 |
|  | *Spirosoma* | 0.0 | 0.1 | 0.0 |  | 0.0 | 0.0 | 0.0 |  | 0.3 | 0.0 | 0.0 |  | 0.0 | 0.0 | 0.0 |  | 0.0 | 0.0 | 0.0 |
|  | *Mucilaginibacter* | 0.2 | 0.1 | 0.0 |  | 0.0 | 0.0 | 0.0 |  | 0.3 | 0.0 | 0.0 |  | 0.0 | 0.0 | 0.0 |  | 0.3 | 0.0 | 0.4 |
|  | *Pedobacter* | 0.2 | 0.2 | 0.2 |  | 0.4 | 0.0 | 0.0 |  | 0.0 | 0.0 | 0.0 |  | 0.0 | 0.0 | 0.0 |  | 0.3 | 0.3 | 0.0 |
|  | *Sphingobacterium* | 0.0 | 0.0 | 0.0 |  | 0.0 | 0.5 | 0.0 |  | 0.0 | 0.0 | 0.4 |  | 0.6 | 0.0 | 0.0 |  | 0.0 | 0.0 | 0.0 |
|  | *Sphingobacteriaceae_u* | 0.0 | 0.1 | 0.0 |  | 0.0 | 0.0 | 0.0 |  | 0.0 | 0.0 | 0.1 |  | 0.0 | 0.0 | 0.0 |  | 0.0 | 0.3 | 0.0 |
|  | *Sphingobacteriales_u* | 0.2 | 0.2 | 0.4 |  | 0.0 | 0.0 | 0.0 |  | 0.0 | 0.0 | 0.6 |  | 0.0 | 0.0 | 0.0 |  | 0.0 | 0.3 | 0.4 |
|  | *Capnocytophaga* | 0.0 | 0.0 | 0.0 |  | 0.0 | 0.0 | 0.0 |  | 0.0 | 0.0 | 0.0 |  | 0.0 | 0.0 | 0.0 |  | 0.0 | 0.3 | 0.0 |
|  | *Chryseobacterium* | 1.1 | 0.6 | 1.0 |  | 1.5 | 2.8 | 0.6 |  | 1.3 | 1.7 | 0.3 |  | 1.1 | 0.5 | 2.5 |  | 2.4 | 1.7 | 0.8 |
|  | *Cloacibacterium* | 0.0 | 0.1 | 0.0 |  | 0.0 | 0.0 | 0.0 |  | 0.0 | 0.6 | 0.1 |  | 0.0 | 0.0 | 0.0 |  | 0.0 | 0.0 | 0.0 |
|  | *Elizabethkingia* | 0.0 | 0.1 | 0.0 |  | 0.0 | 0.0 | 0.0 |  | 0.0 | 0.0 | 0.0 |  | 0.0 | 0.0 | 0.0 |  | 0.0 | 0.0 | 0.4 |
|  | *Empedobacter* | 0.0 | 0.0 | 0.2 |  | 0.0 | 0.0 | 0.0 |  | 0.0 | 0.0 | 0.1 |  | 0.0 | 0.0 | 0.0 |  | 0.0 | 0.0 | 0.0 |
|  | *Flavobacterium* | 0.0 | 0.2 | 0.2 |  | 1.5 | 2.3 | 0.0 |  | 0.3 | 1.7 | 0.1 |  | 4.0 | 1.4 | 0.8 |  | 1.5 | 0.0 | 0.0 |
|  | *Soonwooa* | 0.0 | 0.0 | 0.0 |  | 0.0 | 0.0 | 0.0 |  | 0.0 | 0.0 | 0.1 |  | 0.0 | 0.0 | 0.0 |  | 0.0 | 0.0 | 0.0 |
|  | *Flavobacteriaceae_u* | 0.2 | 1.0 | 0.8 |  | 0.4 | 0.0 | 0.0 |  | 0.0 | 0.0 | 0.3 |  | 0.0 | 0.0 | 0.0 |  | 0.0 | 0.3 | 0.8 |
|  | *Bacteroidetes_u* | 9.8 | 8.8 | 10.9 |  | 3.8 | 0.0 | 6.4 |  | 0.6 | 1.7 | 12.3 |  | 1.1 | 0.0 | 1.7 |  | 0.9 | 1.0 | 1.9 |
| *Deinococcus-Thermus* | *Thermus* | 0.0 | 0.1 | 0.2 |  | 0.0 | 0.0 | 0.0 |  | 0.0 | 0.0 | 0.0 |  | 0.0 | 0.0 | 0.8 |  | 0.0 | 0.0 | 0.0 |
| *Fibrobacteres* | *Fibrobacter* | 0.0 | 0.0 | 0.2 |  | 0.0 | 0.0 | 0.0 |  | 0.0 | 0.0 | 0.1 |  | 0.0 | 0.0 | 0.0 |  | 0.0 | 0.0 | 0.0 |
| *Fusobacteria* | *Leptotrichia* | 0.2 | 0.0 | 0.0 |  | 0.0 | 0.0 | 0.0 |  | 0.0 | 0.0 | 0.0 |  | 0.0 | 0.0 | 0.0 |  | 0.0 | 0.0 | 0.0 |
|  | *Leptotrichiaceae_u* | 0.0 | 0.1 | 0.0 |  | 0.0 | 0.0 | 0.0 |  | 0.0 | 0.0 | 0.0 |  | 0.0 | 0.0 | 0.0 |  | 0.0 | 0.0 | 0.0 |
| *Gemmatimonadetes* | *Gemmatimonas* | 0.0 | 0.0 | 0.0 |  | 0.0 | 0.0 | 0.0 |  | 0.3 | 0.0 | 0.0 |  | 0.0 | 0.0 | 0.0 |  | 0.0 | 0.3 | 0.0 |
| *Planctomycetes* | *Planctomycetaceae_u* | 0.0 | 0.0 | 0.0 |  | 0.0 | 0.0 | 0.0 |  | 0.3 | 0.0 | 0.0 |  | 0.0 | 0.0 | 0.0 |  | 0.0 | 0.0 | 0.0 |
| *Proteobacteria* | *Alcaligenes* | 0.0 | 0.1 | 0.0 |  | 0.0 | 0.0 | 0.0 |  | 0.0 | 0.0 | 0.0 |  | 0.0 | 0.0 | 0.0 |  | 0.0 | 0.0 | 0.0 |
|  | *Oligella* | 0.4 | 0.5 | 0.6 |  | 0.0 | 0.0 | 1.2 |  | 0.0 | 0.0 | 0.4 |  | 0.0 | 0.0 | 0.0 |  | 0.0 | 0.0 | 1.1 |
|  | *Paenalcaligenes* | 0.0 | 0.0 | 0.8 |  | 0.0 | 0.0 | 0.6 |  | 0.0 | 0.0 | 0.0 |  | 0.0 | 0.0 | 0.0 |  | 0.0 | 0.0 | 0.0 |
|  | *Alcaligenaceae_u* | 0.0 | 0.0 | 0.0 |  | 0.0 | 0.0 | 0.0 |  | 0.0 | 0.0 | 0.4 |  | 0.0 | 0.0 | 0.0 |  | 0.0 | 0.0 | 0.0 |
|  | *Burkholderia* | 0.2 | 0.2 | 0.2 |  | 0.4 | 0.0 | 0.0 |  | 0.3 | 0.0 | 0.0 |  | 0.0 | 0.0 | 0.0 |  | 0.3 | 0.3 | 0.4 |
|  | *Cupriavidus* | 0.0 | 0.0 | 0.0 |  | 0.0 | 0.0 | 0.0 |  | 0.0 | 0.0 | 0.1 |  | 0.0 | 0.0 | 0.0 |  | 0.0 | 0.0 | 0.4 |
|  | *Ralstonia* | 0.4 | 0.2 | 0.2 |  | 0.4 | 0.9 | 0.0 |  | 1.0 | 0.6 | 0.1 |  | 0.6 | 0.5 | 0.0 |  | 0.9 | 1.0 | 0.4 |
|  | *Burkholderiaceae_u* | 0.0 | 0.0 | 0.0 |  | 0.0 | 0.0 | 0.0 |  | 0.3 | 0.0 | 0.0 |  | 0.0 | 0.0 | 0.0 |  | 0.0 | 0.0 | 0.0 |
|  | *Aquabacterium* | 0.2 | 0.1 | 0.0 |  | 0.4 | 0.0 | 0.0 |  | 0.6 | 0.0 | 0.1 |  | 0.0 | 0.0 | 0.0 |  | 0.3 | 0.3 | 0.0 |
|  | *Tepidimonas* | 0.2 | 0.0 | 0.2 |  | 0.0 | 0.0 | 0.0 |  | 0.0 | 0.0 | 0.1 |  | 0.6 | 0.0 | 0.0 |  | 0.0 | 0.0 | 0.0 |
|  | *Burkholderiales_incertae_sedis* | 0.2 | 0.1 | 0.2 |  | 0.4 | 0.5 | 0.6 |  | 0.6 | 0.6 | 0.1 |  | 0.0 | 0.0 | 0.0 |  | 0.3 | 0.3 | 0.0 |
|  | *Acidovorax* | 0.2 | 0.1 | 0.2 |  | 0.0 | 0.0 | 0.6 |  | 0.3 | 0.0 | 0.1 |  | 0.0 | 0.0 | 0.8 |  | 0.0 | 0.0 | 0.0 |
|  | *Comamonas* | 0.2 | 0.2 | 0.2 |  | 0.0 | 0.5 | 0.6 |  | 0.0 | 0.6 | 0.0 |  | 0.6 | 0.5 | 0.0 |  | 0.3 | 0.3 | 0.0 |
|  | *Curvibacter* | 0.7 | 0.6 | 0.2 |  | 0.8 | 0.5 | 0.0 |  | 2.6 | 0.6 | 0.0 |  | 0.6 | 0.5 | 0.8 |  | 0.6 | 0.3 | 0.4 |
|  | *Delftia* | 0.4 | 0.1 | 0.2 |  | 0.4 | 0.5 | 0.6 |  | 1.3 | 0.6 | 0.3 |  | 0.6 | 0.5 | 0.0 |  | 0.6 | 0.7 | 0.8 |
|  | *Diaphorobacter* | 0.0 | 0.0 | 0.2 |  | 0.0 | 0.0 | 0.0 |  | 0.3 | 0.0 | 0.1 |  | 0.0 | 0.0 | 0.0 |  | 0.3 | 0.0 | 0.4 |
|  | *Pelomonas* | 0.2 | 0.2 | 0.0 |  | 0.0 | 0.0 | 0.0 |  | 0.3 | 0.0 | 0.3 |  | 0.0 | 0.0 | 0.0 |  | 0.0 | 0.7 | 0.0 |
|  | *Ramlibacter* | 0.2 | 0.0 | 0.0 |  | 0.0 | 0.0 | 0.0 |  | 0.0 | 0.0 | 0.0 |  | 0.0 | 0.0 | 0.0 |  | 0.0 | 0.0 | 0.0 |
|  | *Variovorax* | 0.2 | 0.1 | 0.0 |  | 0.0 | 0.0 | 0.0 |  | 0.3 | 0.0 | 0.0 |  | 0.6 | 0.0 | 0.0 |  | 0.3 | 0.3 | 0.4 |
|  | *Comamonadaceae_u* | 1.4 | 0.5 | 0.0 |  | 0.0 | 0.0 | 0.6 |  | 1.6 | 0.0 | 0.0 |  | 0.0 | 0.0 | 0.0 |  | 1.2 | 0.3 | 0.0 |
|  | *Herbaspirillum* | 0.2 | 0.1 | 0.0 |  | 0.4 | 0.0 | 0.0 |  | 0.3 | 0.0 | 0.0 |  | 0.0 | 0.0 | 0.0 |  | 0.3 | 0.3 | 0.0 |
|  | *Herminiimonas* | 0.0 | 0.0 | 0.0 |  | 0.0 | 0.0 | 0.0 |  | 0.0 | 0.0 | 0.0 |  | 0.0 | 0.0 | 0.0 |  | 0.0 | 0.0 | 0.4 |
|  | *Janthinobacterium* | 0.0 | 0.1 | 0.4 |  | 0.8 | 1.4 | 0.6 |  | 1.0 | 0.6 | 0.1 |  | 0.6 | 1.0 | 0.8 |  | 0.9 | 2.1 | 0.8 |
|  | *Massilia* | 0.4 | 0.4 | 0.0 |  | 0.0 | 0.0 | 0.0 |  | 1.0 | 0.6 | 0.3 |  | 0.0 | 0.0 | 0.0 |  | 0.6 | 0.3 | 0.4 |
|  | *Naxibacter* | 0.0 | 0.0 | 0.0 |  | 0.0 | 0.0 | 0.0 |  | 0.0 | 0.0 | 0.1 |  | 0.0 | 0.0 | 0.0 |  | 0.0 | 0.0 | 0.4 |
|  | *Undibacterium* | 0.5 | 0.6 | 0.2 |  | 0.4 | 0.5 | 0.6 |  | 0.6 | 0.6 | 0.1 |  | 0.0 | 0.5 | 0.0 |  | 0.6 | 0.3 | 0.4 |
|  | *Oxalobacteraceae_u* | 0.4 | 0.4 | 0.0 |  | 0.0 | 0.0 | 0.6 |  | 0.3 | 0.6 | 0.3 |  | 0.0 | 0.0 | 0.0 |  | 0.3 | 1.4 | 0.8 |
|  | *Parasutterella* | 0.0 | 0.5 | 0.0 |  | 0.0 | 0.0 | 0.0 |  | 0.0 | 0.0 | 0.1 |  | 0.0 | 0.0 | 0.0 |  | 0.0 | 0.0 | 0.4 |
|  | *Burkholderiales_u* | 0.0 | 0.2 | 0.0 |  | 0.0 | 0.0 | 0.0 |  | 0.6 | 0.0 | 0.0 |  | 0.0 | 0.0 | 0.0 |  | 0.3 | 0.3 | 0.4 |
|  | *Petrobacter* | 0.0 | 0.0 | 0.0 |  | 0.0 | 0.0 | 0.0 |  | 0.0 | 0.0 | 0.1 |  | 0.0 | 0.0 | 0.0 |  | 0.0 | 0.0 | 0.0 |
|  | *Methylotenera* | 0.2 | 0.1 | 0.0 |  | 0.0 | 0.0 | 0.0 |  | 0.3 | 0.0 | 0.0 |  | 0.0 | 0.0 | 0.0 |  | 0.0 | 0.0 | 0.0 |
|  | *Kingella* | 0.2 | 0.2 | 0.0 |  | 0.0 | 0.0 | 0.0 |  | 0.3 | 0.0 | 0.1 |  | 0.0 | 0.0 | 0.0 |  | 0.3 | 0.0 | 0.0 |
|  | *Neisseria* | 0.0 | 0.1 | 0.0 |  | 0.0 | 0.5 | 0.0 |  | 0.0 | 0.0 | 0.0 |  | 0.0 | 0.0 | 0.0 |  | 0.0 | 0.0 | 0.0 |
|  | *Dechloromonas* | 0.0 | 0.0 | 0.0 |  | 0.0 | 0.0 | 0.0 |  | 0.0 | 0.0 | 0.1 |  | 0.0 | 0.0 | 0.0 |  | 0.0 | 0.0 | 0.0 |
|  | *Thauera* | 0.0 | 0.0 | 0.0 |  | 0.0 | 0.0 | 0.0 |  | 0.3 | 0.0 | 0.0 |  | 0.0 | 0.0 | 0.0 |  | 0.0 | 0.0 | 0.0 |
|  | *Betaproteobacteria_u* | 0.0 | 0.1 | 0.2 |  | 0.0 | 0.0 | 0.0 |  | 0.0 | 0.0 | 0.3 |  | 0.0 | 0.0 | 0.0 |  | 0.3 | 0.0 | 0.0 |
|  | *Desulfobulbus* | 0.0 | 0.1 | 0.2 |  | 0.0 | 0.0 | 0.0 |  | 0.0 | 0.0 | 0.0 |  | 0.0 | 0.0 | 0.0 |  | 0.0 | 0.0 | 0.0 |
|  | *Desulfovibrionaceae_u* | 0.0 | 0.0 | 0.0 |  | 0.0 | 0.0 | 0.0 |  | 0.0 | 0.0 | 0.1 |  | 0.0 | 0.0 | 0.0 |  | 0.0 | 0.0 | 0.0 |
|  | *Desulfuromonas* | 0.0 | 0.0 | 0.0 |  | 0.0 | 0.0 | 0.6 |  | 0.0 | 0.0 | 0.0 |  | 0.0 | 0.0 | 0.0 |  | 0.0 | 0.0 | 0.0 |
|  | *Cystobacter* | 0.0 | 0.1 | 0.0 |  | 0.0 | 0.0 | 0.0 |  | 0.0 | 0.0 | 0.0 |  | 0.0 | 0.0 | 0.0 |  | 0.0 | 0.0 | 0.0 |
|  | *Cystobacteraceae_u* | 0.0 | 0.0 | 0.0 |  | 0.0 | 0.0 | 0.0 |  | 0.0 | 0.0 | 0.0 |  | 0.0 | 0.0 | 0.0 |  | 0.3 | 0.0 | 0.0 |
|  | *Nannocystaceae_u* | 0.0 | 0.1 | 0.0 |  | 0.0 | 0.0 | 0.0 |  | 0.0 | 0.0 | 0.0 |  | 0.0 | 0.0 | 0.0 |  | 0.0 | 0.3 | 0.0 |
|  | *Polyangiaceae_u* | 0.2 | 0.5 | 0.0 |  | 0.0 | 0.0 | 0.0 |  | 1.0 | 0.0 | 0.1 |  | 0.0 | 0.0 | 0.0 |  | 0.0 | 0.0 | 0.0 |
|  | *Myxococcales_u* | 0.4 | 0.1 | 0.0 |  | 0.0 | 0.0 | 0.0 |  | 0.0 | 0.0 | 0.1 |  | 0.0 | 0.0 | 0.0 |  | 0.0 | 0.0 | 0.0 |
|  | *Deltaproteobacteria_u* | 0.0 | 0.0 | 0.0 |  | 0.0 | 0.0 | 0.0 |  | 0.0 | 0.0 | 0.1 |  | 0.0 | 0.0 | 0.0 |  | 0.3 | 0.0 | 0.0 |
|  | *Campylobacter* | 0.2 | 0.1 | 0.8 |  | 0.0 | 0.0 | 0.6 |  | 0.0 | 0.0 | 0.7 |  | 0.0 | 0.0 | 0.0 |  | 0.0 | 0.0 | 0.4 |
|  | *Enterobacter* | 0.0 | 0.0 | 0.0 |  | 0.0 | 0.0 | 0.0 |  | 0.3 | 0.0 | 0.1 |  | 0.0 | 0.0 | 0.0 |  | 0.3 | 0.3 | 0.0 |
|  | *Erwinia* | 0.0 | 0.0 | 0.0 |  | 0.4 | 0.0 | 0.0 |  | 0.0 | 0.0 | 0.0 |  | 0.0 | 0.0 | 0.0 |  | 0.0 | 0.0 | 0.0 |
|  | *Escherichia_Shigella* | 0.0 | 0.1 | 0.2 |  | 0.0 | 0.0 | 0.0 |  | 0.0 | 0.0 | 0.0 |  | 0.0 | 0.0 | 0.0 |  | 0.0 | 0.0 | 0.0 |
|  | *Hafnia* | 0.2 | 0.1 | 0.2 |  | 0.4 | 0.5 | 0.0 |  | 0.3 | 0.6 | 0.1 |  | 0.6 | 0.5 | 0.8 |  | 0.9 | 1.7 | 1.1 |
|  | *Kluyvera* | 0.0 | 0.0 | 0.2 |  | 0.0 | 0.5 | 0.0 |  | 0.0 | 0.6 | 0.0 |  | 0.0 | 0.5 | 0.0 |  | 0.3 | 0.3 | 0.4 |
|  | *Pantoea* | 0.4 | 0.1 | 0.0 |  | 0.4 | 0.0 | 0.0 |  | 0.3 | 0.0 | 0.1 |  | 0.0 | 0.0 | 0.0 |  | 0.0 | 0.3 | 0.4 |
|  | *Providencia* | 0.0 | 0.0 | 0.2 |  | 0.0 | 0.0 | 0.0 |  | 0.0 | 0.6 | 0.0 |  | 0.0 | 0.0 | 0.0 |  | 0.3 | 0.3 | 0.4 |
|  | *Serratia* | 0.0 | 0.1 | 1.6 |  | 2.7 | 1.4 | 0.0 |  | 0.6 | 1.7 | 0.4 |  | 2.3 | 1.4 | 1.7 |  | 3.0 | 4.2 | 0.8 |
|  | *Yersinia* | 0.0 | 0.2 | 1.4 |  | 3.1 | 1.8 | 0.0 |  | 1.0 | 2.9 | 0.0 |  | 3.4 | 2.9 | 0.8 |  | 3.3 | 2.8 | 1.5 |
|  | *Enterobacteriaceae_u* | 1.6 | 0.9 | 9.5 |  | 16.9 | 8.7 | 1.8 |  | 6.2 | 18.9 | 2.0 |  | 15.3 | 12.0 | 9.1 |  | 18.5 | 25.0 | 6.8 |
|  | *Aeromonas* | 0.0 | 0.0 | 0.0 |  | 0.0 | 0.0 | 0.0 |  | 0.0 | 0.0 | 0.0 |  | 0.0 | 0.0 | 0.0 |  | 0.6 | 0.0 | 0.0 |
|  | *Succinivibrionaceae_u* | 0.2 | 0.1 | 0.0 |  | 0.0 | 0.0 | 0.0 |  | 0.0 | 0.0 | 0.1 |  | 0.0 | 0.0 | 0.0 |  | 0.0 | 0.0 | 0.0 |
|  | *Pseudoalteromonas* | 0.0 | 0.0 | 0.0 |  | 0.0 | 0.0 | 0.0 |  | 0.0 | 0.0 | 0.0 |  | 0.0 | 0.0 | 0.0 |  | 0.0 | 0.0 | 0.4 |
|  | *Ectothiorhodospiraceae_u* | 0.2 | 0.0 | 0.0 |  | 0.0 | 0.0 | 0.0 |  | 0.0 | 0.0 | 0.0 |  | 0.0 | 0.0 | 0.0 |  | 0.0 | 0.0 | 0.0 |
|  | *Legionella* | 0.0 | 0.0 | 0.0 |  | 0.0 | 0.0 | 0.0 |  | 0.3 | 0.0 | 0.0 |  | 0.0 | 0.0 | 0.0 |  | 0.0 | 0.0 | 0.0 |
|  | *Halomonas* | 0.0 | 0.4 | 0.0 |  | 0.0 | 0.0 | 0.0 |  | 0.0 | 0.0 | 0.1 |  | 0.0 | 0.0 | 0.0 |  | 0.0 | 0.0 | 0.0 |
|  | *Pasteurellaceae_u* | 0.4 | 0.0 | 0.0 |  | 0.0 | 0.0 | 0.0 |  | 0.0 | 0.0 | 0.1 |  | 0.0 | 0.0 | 0.0 |  | 0.3 | 0.0 | 0.0 |
|  | *Acinetobacter* | 5.5 | 3.5 | 3.7 |  | 13.5 | 17.4 | 6.4 |  | 6.8 | 8.0 | 6.0 |  | 15.9 | 15.8 | 6.6 |  | 9.7 | 9.0 | 4.9 |
|  | *Enhydrobacter* | 0.4 | 0.4 | 0.4 |  | 0.0 | 0.5 | 0.6 |  | 0.6 | 0.6 | 0.3 |  | 0.0 | 0.5 | 0.8 |  | 0.6 | 0.7 | 0.4 |
|  | *Moraxella* | 0.0 | 0.1 | 0.0 |  | 0.0 | 0.0 | 0.0 |  | 0.0 | 0.0 | 0.0 |  | 0.0 | 0.0 | 0.0 |  | 0.0 | 0.0 | 0.0 |
|  | *Psychrobacter* | 0.7 | 0.7 | 1.4 |  | 0.4 | 0.5 | 1.8 |  | 1.0 | 0.6 | 2.0 |  | 0.6 | 0.0 | 0.8 |  | 0.6 | 0.3 | 1.1 |
|  | *Moraxellaceae_u* | 0.0 | 0.1 | 0.2 |  | 0.0 | 0.0 | 0.0 |  | 0.3 | 0.0 | 0.0 |  | 0.6 | 0.5 | 0.0 |  | 0.0 | 0.0 | 0.0 |
|  | *Pseudomonas* | 3.4 | 3.0 | 4.9 |  | 23.8 | 33.0 | 32.2 |  | 17.9 | 37.7 | 8.7 |  | 33.0 | 40.2 | 47.1 |  | 13.0 | 9.7 | 30.2 |
|  | *Rhizobacter* | 0.0 | 0.1 | 0.0 |  | 0.0 | 0.0 | 0.0 |  | 0.0 | 0.0 | 0.1 |  | 0.0 | 0.0 | 0.0 |  | 0.0 | 0.0 | 0.0 |
|  | *Pseudomonadaceae_u* | 0.0 | 0.0 | 0.0 |  | 0.4 | 0.5 | 0.0 |  | 0.0 | 0.0 | 0.0 |  | 0.6 | 1.0 | 0.8 |  | 0.6 | 0.0 | 0.0 |
|  | *Pseudomonadales_u* | 0.0 | 0.1 | 0.0 |  | 5.0 | 8.7 | 0.6 |  | 0.3 | 0.6 | 0.6 |  | 1.7 | 6.2 | 0.0 |  | 0.6 | 1.0 | 0.0 |
|  | *Methylophaga* | 0.0 | 0.1 | 0.0 |  | 0.0 | 0.0 | 0.0 |  | 0.0 | 0.0 | 0.0 |  | 0.0 | 0.0 | 0.0 |  | 0.0 | 0.0 | 0.0 |
|  | *Steroidobacter* | 0.2 | 0.1 | 0.0 |  | 0.0 | 0.0 | 0.0 |  | 0.0 | 0.6 | 0.0 |  | 0.0 | 0.0 | 0.0 |  | 0.0 | 0.0 | 0.0 |
|  | *Luteibacter* | 0.2 | 0.0 | 0.0 |  | 0.0 | 0.0 | 0.6 |  | 0.0 | 0.0 | 0.0 |  | 0.0 | 0.0 | 0.0 |  | 0.0 | 0.0 | 0.0 |
|  | *Lysobacter* | 0.2 | 0.0 | 0.0 |  | 0.0 | 0.0 | 0.0 |  | 0.3 | 0.0 | 0.0 |  | 0.0 | 0.0 | 0.0 |  | 0.0 | 0.0 | 0.0 |
|  | *Pseudofulvimonas* | 0.0 | 0.1 | 0.0 |  | 0.0 | 0.0 | 0.0 |  | 0.0 | 0.0 | 0.0 |  | 0.0 | 0.0 | 0.0 |  | 0.0 | 0.0 | 0.0 |
|  | *Pseudoxanthomonas* | 0.0 | 0.1 | 0.0 |  | 0.0 | 0.0 | 0.0 |  | 0.0 | 0.0 | 0.1 |  | 0.0 | 0.0 | 0.0 |  | 0.0 | 0.0 | 0.0 |
|  | *Stenotrophomonas* | 0.5 | 0.1 | 0.6 |  | 0.0 | 0.0 | 0.6 |  | 0.3 | 0.0 | 0.3 |  | 0.0 | 0.0 | 0.0 |  | 0.3 | 0.0 | 0.4 |
|  | *Thermomonas* | 0.0 | 0.1 | 0.2 |  | 0.0 | 0.0 | 0.0 |  | 0.0 | 0.0 | 0.0 |  | 0.0 | 0.0 | 0.0 |  | 0.0 | 0.0 | 0.0 |
|  | *Xanthomonas* | 0.0 | 0.0 | 0.0 |  | 0.0 | 0.0 | 0.0 |  | 0.0 | 0.0 | 0.1 |  | 0.0 | 0.0 | 0.0 |  | 0.0 | 0.0 | 0.0 |
|  | *Xanthomonadaceae_u* | 1.6 | 1.1 | 1.2 |  | 0.4 | 0.5 | 0.6 |  | 3.2 | 0.6 | 1.1 |  | 0.6 | 0.5 | 0.8 |  | 1.2 | 1.4 | 2.3 |
|  | *Gammaproteobacteria_u* | 0.2 | 0.6 | 0.8 |  | 3.5 | 4.6 | 0.0 |  | 1.3 | 0.6 | 0.6 |  | 1.1 | 1.9 | 0.0 |  | 2.1 | 1.7 | 0.4 |
|  | *Proteobacteria_u* | 0.0 | 0.5 | 0.2 |  | 0.0 | 0.5 | 1.2 |  | 0.6 | 0.0 | 0.4 |  | 0.0 | 0.0 | 0.0 |  | 0.9 | 0.0 | 0.0 |
| *Spirochaetes* | *Treponema* | 0.0 | 0.1 | 0.4 |  | 0.0 | 0.0 | 0.0 |  | 0.0 | 0.0 | 0.3 |  | 0.0 | 0.0 | 0.0 |  | 0.0 | 0.0 | 0.0 |
|  | *Spirochaetales_u* | 0.0 | 0.1 | 0.0 |  | 0.0 | 0.0 | 0.0 |  | 0.0 | 0.0 | 0.0 |  | 0.0 | 0.0 | 0.0 |  | 0.0 | 0.0 | 0.0 |
| *Tenericutes* | *Acholeplasma* | 0.0 | 0.0 | 0.0 |  | 0.0 | 0.0 | 0.0 |  | 0.0 | 0.0 | 0.1 |  | 0.0 | 0.0 | 0.0 |  | 0.0 | 0.0 | 0.0 |
|  | *Anaeroplasma* | 0.2 | 0.0 | 0.6 |  | 0.0 | 0.0 | 0.0 |  | 0.0 | 0.0 | 0.1 |  | 0.0 | 0.0 | 0.0 |  | 0.0 | 0.0 | 0.0 |
|  | *Mollicutes_u* | 0.0 | 0.0 | 0.0 |  | 0.0 | 0.0 | 0.0 |  | 0.0 | 0.0 | 0.1 |  | 0.0 | 0.0 | 0.0 |  | 0.0 | 0.0 | 0.0 |
| *Firmicutes* | *Bacillus* | 0.2 | 0.2 | 0.0 |  | 0.0 | 0.0 | 0.0 |  | 0.3 | 0.0 | 0.1 |  | 0.0 | 0.5 | 0.0 |  | 0.6 | 0.0 | 0.0 |
|  | *Bacillaceae_1_u* | 0.0 | 0.1 | 0.0 |  | 0.0 | 0.0 | 0.0 |  | 0.0 | 0.0 | 0.0 |  | 0.0 | 0.0 | 0.0 |  | 0.0 | 0.0 | 0.0 |
|  | *Gemella* | 0.0 | 0.0 | 0.0 |  | 0.0 | 0.0 | 0.0 |  | 0.3 | 0.0 | 0.1 |  | 0.0 | 0.0 | 0.0 |  | 0.0 | 0.0 | 0.0 |
|  | *Exiguobacterium* | 0.2 | 0.0 | 0.0 |  | 0.0 | 0.0 | 0.0 |  | 0.3 | 0.0 | 0.0 |  | 0.0 | 0.0 | 0.0 |  | 0.0 | 0.0 | 0.0 |
|  | *Brochothrix* | 0.2 | 0.1 | 0.4 |  | 0.4 | 0.0 | 0.0 |  | 0.3 | 1.1 | 0.0 |  | 0.0 | 0.0 | 1.7 |  | 0.3 | 1.0 | 0.4 |
|  | *Paenibacillaceae_1_u* | 0.0 | 0.0 | 0.0 |  | 0.0 | 0.0 | 0.0 |  | 0.0 | 0.0 | 0.0 |  | 0.0 | 0.0 | 0.0 |  | 0.0 | 0.0 | 0.4 |
|  | *Caryophanon* | 0.0 | 0.0 | 0.0 |  | 0.0 | 0.0 | 0.0 |  | 0.0 | 0.0 | 0.4 |  | 0.0 | 0.0 | 0.0 |  | 0.0 | 0.0 | 0.0 |
|  | *Planococcaceae_u* | 0.0 | 0.2 | 0.0 |  | 0.0 | 0.0 | 0.6 |  | 0.0 | 0.0 | 0.0 |  | 0.0 | 0.0 | 0.0 |  | 0.0 | 0.0 | 0.0 |
|  | *Jeotgalicoccus* | 2.0 | 1.0 | 0.2 |  | 0.0 | 0.0 | 0.0 |  | 0.0 | 0.0 | 0.0 |  | 0.0 | 0.0 | 0.0 |  | 0.0 | 0.0 | 0.0 |
|  | *Macrococcus* | 0.7 | 0.0 | 0.0 |  | 0.8 | 0.0 | 0.6 |  | 0.0 | 0.0 | 0.1 |  | 0.6 | 0.0 | 0.0 |  | 0.0 | 0.0 | 0.4 |
|  | *Nosocomiicoccus* | 0.0 | 0.0 | 0.2 |  | 0.0 | 0.0 | 0.6 |  | 0.0 | 0.0 | 0.1 |  | 0.0 | 0.0 | 0.0 |  | 0.0 | 0.3 | 0.0 |
|  | *Staphylococcus* | 1.8 | 1.0 | 1.2 |  | 0.0 | 0.5 | 1.8 |  | 2.3 | 0.6 | 1.3 |  | 0.6 | 0.5 | 0.0 |  | 1.5 | 0.7 | 1.9 |
|  | *Bacillales_u* | 0.0 | 0.0 | 0.0 |  | 0.0 | 0.0 | 0.0 |  | 0.0 | 0.0 | 0.0 |  | 0.0 | 0.0 | 0.0 |  | 0.0 | 0.0 | 0.4 |
|  | *Aerococcus* | 2.7 | 1.2 | 1.7 |  | 0.8 | 0.5 | 1.8 |  | 0.0 | 0.0 | 1.1 |  | 0.0 | 0.5 | 1.7 |  | 0.9 | 0.0 | 1.9 |
|  | *Facklamia* | 0.7 | 1.2 | 0.4 |  | 0.0 | 0.0 | 0.0 |  | 0.0 | 0.0 | 0.6 |  | 0.0 | 0.0 | 0.0 |  | 0.0 | 0.0 | 0.8 |
|  | *Globicatella* | 0.4 | 0.1 | 0.0 |  | 0.0 | 0.0 | 0.0 |  | 0.0 | 0.0 | 0.1 |  | 0.0 | 0.0 | 0.0 |  | 0.0 | 0.0 | 0.4 |
|  | *Ignavigranum* | 0.0 | 0.1 | 0.0 |  | 0.0 | 0.0 | 0.0 |  | 0.0 | 0.0 | 0.0 |  | 0.0 | 0.0 | 0.0 |  | 0.0 | 0.0 | 0.0 |
|  | *Aerococcaceae_u* | 0.5 | 0.5 | 0.4 |  | 0.0 | 0.0 | 0.0 |  | 0.0 | 0.0 | 0.4 |  | 0.0 | 0.0 | 0.0 |  | 0.0 | 0.0 | 0.0 |
|  | *Atopostipes* | 0.4 | 0.6 | 0.0 |  | 0.0 | 0.0 | 0.0 |  | 0.0 | 0.0 | 0.1 |  | 0.0 | 0.0 | 0.0 |  | 0.0 | 0.0 | 0.0 |
|  | *Carnobacterium* | 0.0 | 0.0 | 0.2 |  | 0.0 | 0.0 | 0.0 |  | 0.0 | 1.1 | 0.3 |  | 0.0 | 0.0 | 1.7 |  | 0.0 | 1.0 | 0.4 |
|  | *Granulicatella* | 0.2 | 0.0 | 0.0 |  | 0.0 | 0.0 | 0.0 |  | 0.0 | 0.0 | 0.1 |  | 0.0 | 0.0 | 0.0 |  | 0.0 | 0.0 | 0.0 |
|  | *Trichococcus* | 0.0 | 0.1 | 0.0 |  | 0.0 | 0.0 | 0.0 |  | 0.0 | 0.0 | 0.0 |  | 0.0 | 0.0 | 0.0 |  | 0.0 | 0.0 | 0.0 |
|  | *Carnobacteriaceae_u* | 1.2 | 1.1 | 1.0 |  | 0.4 | 0.0 | 0.6 |  | 0.3 | 0.0 | 0.7 |  | 0.0 | 0.0 | 0.8 |  | 0.6 | 0.0 | 0.8 |
|  | *Enterococcus* | 0.7 | 0.4 | 0.4 |  | 0.8 | 0.5 | 0.6 |  | 1.0 | 1.1 | 0.6 |  | 1.1 | 0.5 | 0.8 |  | 1.2 | 1.4 | 1.5 |
|  | *Lactobacillus* | 0.7 | 0.5 | 0.4 |  | 0.0 | 0.0 | 0.0 |  | 0.6 | 0.0 | 0.7 |  | 0.0 | 0.0 | 0.0 |  | 0.6 | 0.0 | 0.0 |
|  | *Lactobacillaceae_u* | 0.2 | 0.1 | 0.2 |  | 0.0 | 0.0 | 0.0 |  | 0.0 | 0.0 | 0.0 |  | 0.0 | 0.0 | 0.0 |  | 0.0 | 0.0 | 0.8 |
|  | *Leuconostoc* | 0.4 | 0.2 | 0.2 |  | 0.0 | 0.0 | 0.0 |  | 0.6 | 0.6 | 0.1 |  | 0.6 | 0.0 | 0.8 |  | 0.6 | 0.0 | 1.5 |
|  | *Weissella* | 0.0 | 0.1 | 0.0 |  | 0.0 | 0.0 | 0.0 |  | 0.0 | 0.0 | 0.0 |  | 0.0 | 0.0 | 0.0 |  | 0.0 | 0.0 | 0.0 |
|  | *Lactococcus* | 0.5 | 0.4 | 0.2 |  | 2.3 | 0.5 | 1.8 |  | 0.6 | 0.0 | 0.4 |  | 1.7 | 0.0 | 2.5 |  | 0.6 | 0.0 | 1.5 |
|  | *Streptococcus* | 1.4 | 0.6 | 0.6 |  | 0.4 | 0.0 | 0.0 |  | 0.6 | 0.0 | 1.4 |  | 0.0 | 0.0 | 0.0 |  | 0.6 | 0.3 | 1.1 |
|  | *Lactobacillales_u* | 0.9 | 1.1 | 1.6 |  | 0.0 | 0.0 | 1.2 |  | 0.0 | 0.0 | 1.7 |  | 0.6 | 0.0 | 0.8 |  | 0.3 | 0.0 | 1.5 |
|  | *Clostridium_sensu_stricto* | 0.2 | 0.6 | 0.4 |  | 0.0 | 0.0 | 0.6 |  | 0.3 | 0.0 | 0.1 |  | 0.0 | 0.0 | 0.0 |  | 0.0 | 0.0 | 0.8 |
|  | *Clostridiaceae_1_u* | 0.0 | 0.0 | 0.0 |  | 0.0 | 0.0 | 0.0 |  | 0.0 | 0.0 | 0.1 |  | 0.0 | 0.0 | 0.8 |  | 0.0 | 0.0 | 0.0 |
|  | *Anaerococcus* | 0.2 | 0.4 | 0.2 |  | 0.0 | 0.0 | 0.0 |  | 0.3 | 0.0 | 0.3 |  | 0.0 | 0.0 | 0.0 |  | 0.3 | 0.3 | 0.0 |
|  | *Finegoldia* | 0.0 | 0.0 | 0.0 |  | 0.0 | 0.0 | 0.0 |  | 0.0 | 0.0 | 0.1 |  | 0.0 | 0.0 | 0.0 |  | 0.3 | 0.0 | 0.0 |
|  | *Gallicola* | 0.0 | 0.0 | 0.0 |  | 0.0 | 0.0 | 0.0 |  | 0.0 | 0.0 | 0.0 |  | 0.0 | 0.0 | 0.0 |  | 0.3 | 0.0 | 0.0 |
|  | *Helcococcus* | 0.2 | 0.1 | 0.0 |  | 0.0 | 0.0 | 0.0 |  | 0.0 | 0.0 | 0.1 |  | 0.0 | 0.0 | 0.0 |  | 0.0 | 0.0 | 0.0 |
|  | *Peptoniphilus* | 0.0 | 0.1 | 0.0 |  | 0.0 | 0.0 | 0.0 |  | 0.6 | 0.0 | 0.0 |  | 0.0 | 0.0 | 0.0 |  | 0.3 | 0.3 | 0.0 |
|  | *Clostridiales_Incertae_Sedis_XI* | 0.2 | 0.0 | 0.0 |  | 0.0 | 0.0 | 0.0 |  | 0.0 | 0.0 | 0.0 |  | 0.0 | 0.0 | 0.0 |  | 0.0 | 0.0 | 0.0 |
|  | *Guggenheimella* | 0.0 | 0.0 | 0.2 |  | 0.0 | 0.0 | 0.0 |  | 0.0 | 0.0 | 0.0 |  | 0.0 | 0.0 | 0.0 |  | 0.0 | 0.0 | 0.0 |
|  | *Anaerovorax* | 0.2 | 0.0 | 0.0 |  | 0.0 | 0.0 | 0.0 |  | 0.0 | 0.0 | 0.1 |  | 0.0 | 0.0 | 0.0 |  | 0.0 | 0.0 | 0.0 |
|  | *Clostridiales_Incertae_Sedis_XIII* | 0.2 | 0.2 | 0.0 |  | 0.0 | 0.0 | 0.0 |  | 0.0 | 0.0 | 0.1 |  | 0.0 | 0.0 | 0.0 |  | 0.0 | 0.0 | 0.4 |
|  | *Murdochiella* | 0.0 | 0.1 | 0.0 |  | 0.0 | 0.0 | 0.0 |  | 0.0 | 0.0 | 0.0 |  | 0.0 | 0.0 | 0.0 |  | 0.0 | 0.0 | 0.0 |
|  | *Blautia* | 0.0 | 0.0 | 0.2 |  | 0.0 | 0.0 | 0.0 |  | 0.0 | 0.0 | 0.0 |  | 0.0 | 0.0 | 0.0 |  | 0.0 | 0.0 | 0.0 |
|  | *Cellulosilyticum* | 0.0 | 0.1 | 0.6 |  | 0.0 | 0.0 | 0.0 |  | 0.0 | 0.0 | 0.0 |  | 0.0 | 0.0 | 0.0 |  | 0.0 | 0.0 | 0.0 |
|  | *Clostridium_XlVa* | 0.0 | 0.0 | 0.0 |  | 0.0 | 0.0 | 0.0 |  | 0.0 | 0.0 | 0.1 |  | 0.0 | 0.0 | 0.0 |  | 0.0 | 0.0 | 0.0 |
|  | *Clostridium_XlVb* | 0.4 | 0.4 | 0.0 |  | 0.0 | 0.0 | 0.0 |  | 0.0 | 0.0 | 0.0 |  | 0.0 | 0.0 | 0.0 |  | 0.0 | 0.0 | 0.0 |
|  | *Lachnospiracea_incertae_sedis* | 0.2 | 0.0 | 0.4 |  | 0.0 | 0.0 | 0.0 |  | 0.0 | 0.0 | 0.0 |  | 0.0 | 0.0 | 0.0 |  | 0.0 | 0.0 | 0.0 |
|  | *Robinsoniella* | 0.0 | 0.0 | 0.0 |  | 0.0 | 0.0 | 0.0 |  | 0.0 | 0.0 | 0.0 |  | 0.0 | 0.0 | 0.0 |  | 0.0 | 0.0 | 0.4 |
|  | *Lachnospiraceae_u* | 3.0 | 3.1 | 2.1 |  | 0.4 | 0.0 | 0.0 |  | 0.6 | 0.0 | 1.1 |  | 0.0 | 0.0 | 0.0 |  | 0.6 | 0.3 | 1.9 |
|  | *Peptococcaceae_1_u* | 0.2 | 0.0 | 0.0 |  | 0.0 | 0.0 | 0.0 |  | 0.0 | 0.0 | 0.0 |  | 0.0 | 0.0 | 0.0 |  | 0.0 | 0.0 | 0.0 |
|  | *Clostridium_XI* | 0.7 | 0.7 | 1.0 |  | 0.4 | 0.0 | 1.8 |  | 0.3 | 0.0 | 0.4 |  | 0.0 | 0.0 | 0.0 |  | 0.3 | 0.3 | 0.8 |
|  | *Peptostreptococcaceae_u* | 0.2 | 0.1 | 0.0 |  | 0.0 | 0.0 | 0.6 |  | 0.0 | 0.0 | 0.3 |  | 0.0 | 0.0 | 0.0 |  | 0.0 | 0.0 | 0.0 |
|  | *Clostridium_IV* | 0.0 | 0.0 | 0.0 |  | 0.0 | 0.0 | 0.0 |  | 0.0 | 0.0 | 0.1 |  | 0.0 | 0.0 | 0.0 |  | 0.0 | 0.0 | 0.0 |
|  | *Faecalibacterium* | 0.0 | 0.0 | 0.0 |  | 0.0 | 0.0 | 0.0 |  | 0.0 | 0.6 | 0.0 |  | 0.0 | 0.5 | 0.0 |  | 0.0 | 0.0 | 0.4 |
|  | *Oscillibacter* | 1.1 | 0.7 | 0.6 |  | 0.0 | 0.0 | 0.0 |  | 0.0 | 0.0 | 0.8 |  | 0.0 | 0.0 | 0.0 |  | 0.0 | 0.0 | 0.0 |
|  | *Pseudoflavonifractor* | 0.2 | 0.1 | 0.2 |  | 0.0 | 0.0 | 0.0 |  | 0.0 | 0.0 | 0.3 |  | 0.0 | 0.0 | 0.0 |  | 0.0 | 0.0 | 0.0 |
|  | *Ruminococcus* | 0.0 | 0.0 | 0.2 |  | 0.0 | 0.0 | 0.0 |  | 0.0 | 0.0 | 0.0 |  | 0.0 | 0.0 | 0.0 |  | 0.0 | 0.0 | 0.0 |
|  | *Ruminococcaceae_u* | 4.8 | 6.6 | 3.7 |  | 0.4 | 0.0 | 0.6 |  | 1.6 | 0.0 | 5.0 |  | 0.0 | 0.0 | 0.0 |  | 0.3 | 0.7 | 0.4 |
|  | *Thermohydrogenium* | 0.0 | 0.1 | 0.0 |  | 0.0 | 0.0 | 0.0 |  | 0.0 | 0.0 | 0.0 |  | 0.0 | 0.0 | 0.0 |  | 0.0 | 0.0 | 0.0 |
|  | *Clostridiales_u* | 3.7 | 7.8 | 6.0 |  | 0.0 | 0.0 | 1.2 |  | 0.6 | 0.0 | 4.9 |  | 0.0 | 0.5 | 0.0 |  | 0.3 | 0.0 | 1.5 |
|  | *Clostridia_u* | 0.2 | 0.1 | 0.0 |  | 0.0 | 0.0 | 0.0 |  | 0.0 | 0.0 | 0.1 |  | 0.0 | 0.0 | 0.0 |  | 0.0 | 0.0 | 0.0 |
|  | *Coprobacillus* | 0.0 | 0.0 | 0.0 |  | 0.0 | 0.0 | 0.0 |  | 0.0 | 0.0 | 0.1 |  | 0.0 | 0.0 | 0.0 |  | 0.0 | 0.0 | 0.0 |
|  | *Succiniclasticum* | 0.0 | 0.4 | 0.0 |  | 0.0 | 0.0 | 0.0 |  | 0.0 | 0.0 | 0.0 |  | 0.0 | 0.0 | 0.0 |  | 0.0 | 0.0 | 0.0 |
|  | *Acidaminococcaceae_u* | 0.4 | 0.2 | 0.4 |  | 0.0 | 0.5 | 1.8 |  | 0.0 | 0.0 | 0.1 |  | 0.0 | 0.0 | 0.0 |  | 0.0 | 0.3 | 0.0 |
|  | *Anaerovibrio* | 0.2 | 0.1 | 1.0 |  | 0.0 | 0.0 | 0.0 |  | 0.0 | 0.0 | 0.0 |  | 0.0 | 0.0 | 0.0 |  | 0.0 | 0.0 | 0.0 |
|  | *Dialister* | 0.0 | 0.0 | 0.0 |  | 0.4 | 0.0 | 0.0 |  | 0.0 | 0.0 | 0.0 |  | 0.0 | 0.0 | 0.0 |  | 0.0 | 0.0 | 0.0 |
|  | *Megasphaera* | 0.2 | 0.0 | 0.0 |  | 0.0 | 0.0 | 0.0 |  | 0.3 | 0.0 | 0.0 |  | 0.0 | 0.0 | 0.0 |  | 0.3 | 0.0 | 0.0 |
|  | *Selenomonas* | 0.0 | 0.0 | 0.0 |  | 0.0 | 0.0 | 0.0 |  | 0.6 | 0.0 | 0.0 |  | 0.0 | 0.0 | 0.0 |  | 0.0 | 0.0 | 0.0 |
|  | *Veillonella* | 0.2 | 0.0 | 0.0 |  | 0.0 | 0.0 | 0.0 |  | 0.3 | 0.0 | 0.3 |  | 0.0 | 0.0 | 0.0 |  | 0.0 | 0.0 | 0.0 |
|  | *Veillonellaceae_u* | 0.4 | 0.2 | 0.0 |  | 0.4 | 0.0 | 0.0 |  | 0.0 | 0.6 | 0.0 |  | 0.0 | 0.0 | 0.0 |  | 0.3 | 0.3 | 0.0 |
|  | *Selenomonadales_u* | 0.0 | 0.1 | 0.0 |  | 0.0 | 0.0 | 0.0 |  | 0.0 | 0.6 | 0.0 |  | 0.0 | 0.0 | 0.8 |  | 0.3 | 0.3 | 0.4 |
|  | *Firmicutes_u* | 3.7 | 3.9 | 3.1 |  | 0.0 | 0.0 | 0.6 |  | 0.6 | 0.6 | 4.2 |  | 0.0 | 0.0 | 0.8 |  | 0.0 | 0.0 | 0.4 |
| unclassified | *Bacteria_u* | 2.1 | 4.5 | 4.1 |  | 0.4 | 0.5 | 4.7 |  | 1.6 | 0.6 | 5.3 |  | 2.3 | 1.0 | 0.8 |  | 1.5 | 3.5 | 1.1 |
|  |  |  |  |  |  |  |  |  |  |  |  |  |  |  |  |  |  |  |  |  |

^a^ Genus or highest phylogenetic level into which sequences could be classified using SILVA reference database

u_ unclassified
